# Antibacterial Activity and Membrane-Targeting Mechanism of Aloe-Emodin Against *Staphylococcus epidermidis*

**DOI:** 10.3389/fmicb.2021.621866

**Published:** 2021-08-16

**Authors:** Tao Li, Yan Lu, Hua Zhang, Lei Wang, Ross C. Beier, Yajie Jin, Wenjing Wang, Huanrong Li, Xiaolin Hou

**Affiliations:** ^1^Shanghai Veterinary Research Institute, CAAS, Shanghai, China; ^2^Beijing Key Laboratory of Chinese Veterinary Medicine, Department of Veterinary Medicine, National Demonstration Center for Experimental Animal Education, Beijing University of Agriculture, Beijing, China; ^3^Beijing Huafukang Bioscience Co., Ltd., Beijing, China; ^4^Food and Feed Safety Research Unit, Southern Plains Agricultural Research Center, Agricultural Research Service, United States Department of Agriculture, College Station, TX, United States

**Keywords:** aloe-emodin, *Staphylococcus epidermidis*, membrane target, inhibit, biofilm

## Abstract

The emergence of multidrug-resistant Staphylococcus epidermidis (*S. epidermidis) dwarfs* the current antibiotic development and calls for the discovery of new antibacterial agents. Aloe-emodin is a plant-derived compound that holds promise to battle against these strains. This work reports the antimicrobial activity of aloe-emodin against *S. epidermidis* and other Gram-positive pathogenic species, manifesting minimum inhibitory concentrations (MICs) and minimum bactericidal concentration (MBCs) around 4–32 and 32–128 μg/mL, respectively. For Gram-negative bacteria tested, the MICs and MBCs of aloe-emodin were 128–256 and above 1024 μg/mL, respectively. Aloe-emodin at the MBC for 4 h eradicated 96.9% of *S. epidermidis* cells. Aloe-emodin treatment led to deformities in the morphology of *S. epidermidis* cells and the destroy of the selective permeability of the cell membranes. Analysis of the transcriptional profiles of aloe-emodin-treated cells revealed changes of genes involved in sulfur metabolism, L-lysine and peptidoglycan biosynthesis, and biofilm formation. Aloe-emodin therefore can safely control Gram-positive bacterial infections and proves to target the bacterial outer membrane.

## Introduction

The excessive use of antibiotics as human and veterinary medicines, as well as in animal production, has had significant effects on public health (Economou and Gousia, 2015). The misuse of antibiotics accelerates the mutation−driven production of antimicrobial resistance by selecting for bacteria with resistance determinants and then spreading them both vertically and horizontally. Multidrug-resistant bacteria, or “superbugs,” cause an estimated 700,000 deaths a year worldwide (Anthony et al., 2018). Therefore, the rapid development and dissemination of bacterial resistance to conventional antibiotics have become a global threat to public health.

*Staphylococcus epidermidis* is a Gram-positive bacterium commonly found on the skin and in the mucosa of humans and animals (Kleinschmidt et al., 2015). It is one of the most common causes of bacteremia and sepsis (Otto, 2017; Lam et al., 2018), particularly in individuals with traumatic epidermal injuries, indwelling medical devices (such as catheters and prostheses) (Rupp, 2014), or weakened immune function (Wessels et al., 2018). It has also been shown to cause mastitis in dairy cows, as it is able to penetrate the ulcerated tissue and enter the bloodstream (Zhou et al., 2015). The emergence of drug-resistant strains over the last few decades has posed a challenge for the treatment and control of *S. epidermidis*, as most of the clinical isolates are resistant to β-lactam antibiotics, and some even to glycopeptide antibiotics (Cherifi et al., 2014). As such, there is an urgent need to develop new and effective drugs against resistant *S. epidermidis* strains (Amato et al., 2018; Costa et al., 2018; Medvedec et al., 2018).

**FIGURE 1:**
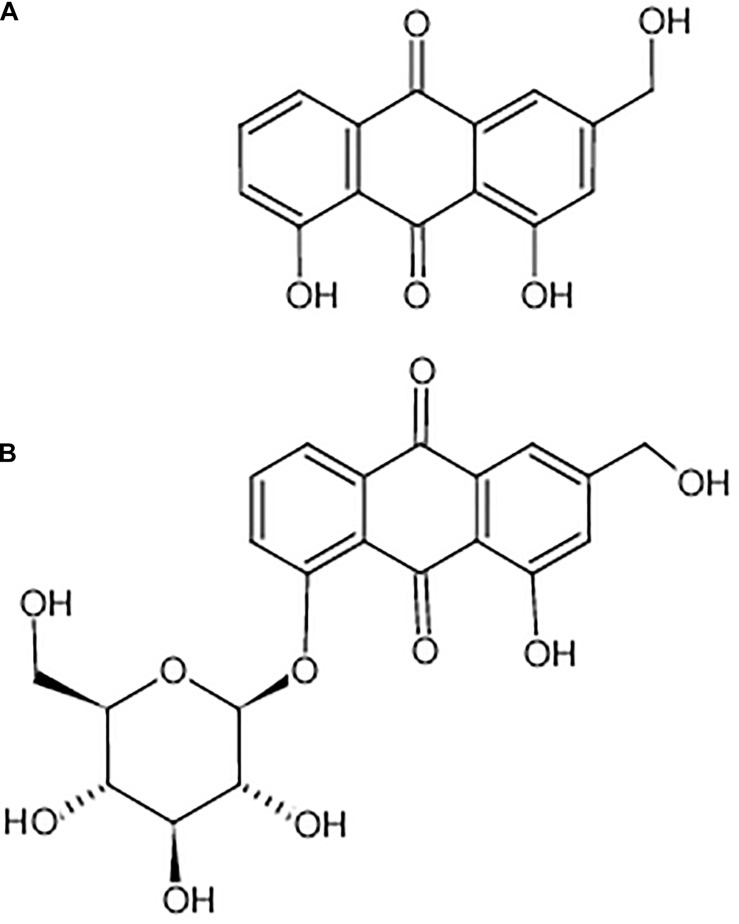
Chemical structure of aloe-emodin. Aloe-emodin is an anthraquinone derivative and is either presented in aloe in free form **(A)** or presented in rhubarb, senna, and aloe in the form of glycosides **(B)**.

Aloe-emodin is a natural anthraquinone derivative and an active ingredient that can be isolated from *Cassia occidentalis*, *Rheum*, *Aloe vera*, and *Polygonum multiflorum Thunb*. Emerging evidence suggests that aloe-emodin exhibits many pharmacological effects (chemical structure shown in Figure 1) (Dong et al., 2020), such as antiviral (Li et al., 2014), anti-inflammatory, and antibacterial effects (Xiang et al., 2017; Jiang et al., 2019), promotory effects on the immune response (Devi et al., 2019), and anti-cancer (Ozenver et al., 2018) effects. Aloe-emodin inhibits *Staphylococcus aureus* biofilm formation by the inhibition of extracellular protein production (Xiang et al., 2017). Aloe-emodin attenuates *S. aureus* pathogenicity by interfering with the oligomerization of α-toxin (Jiang et al., 2019). *Rheum emodi* is an important medicinal herb that has demonstrated antibacterial activity against acute gastroenteritis, and aloe-emodin is one of its active components (Jiang et al., 2018). However, the antibacterial mechanism of aloe-emodin against *S. epidermidis* remains unclear, and is the focus of this study. The reference strain *Staphylococcus aureus* M2 CVCC1882 (MSSA), *Staphylococcus aureus* ATCC 33591 (MRSA), *Staphylococcus aureus* ATCC43300 (MRSA), *Streptococcus pneumoniae* ATCC 49619, *Escherichia coli* BNCC133264, *Escherichia coli* ATCC 25922, and *Pseudomonas. aeruginosa* ATCC 27853 were also used to indicate the antibacterial activity of aloe-emodin on the different sources of bacterial.

## Results

### Antibacterial Activity and Cytotoxicity

**FIGURE 2:**
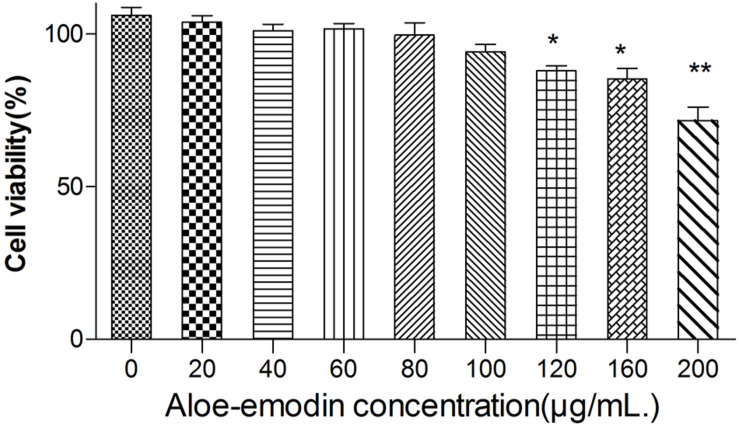
Cytotoxicity of aloe-emodin as determined by the MTT method. Cytotoxicity is shown as the OD value of RAW 264.7 cells under aloe-emodin exposure. Aloe-emodin exhibited cytotoxicity in a dose-dependent manner. High doses (> 100 μg/mL) of aloe-emodin significantly decreased cellular viability, whereas low doses showed no cytotoxicity. Single asterisk (*) indicates *P* < 0.05, and double asterisks (**) indicate *P* < 0.01 vs. non-treatment group.

Among the bacteria tested (Table 1), aloe-emodin has higher minimum inhibitory concentrations (MICs) than vancomycin, oxacillin, and levofloxacin. Its effects are independent of resistance to the other tested drugs. Its activity against Gram-positive bacteria is higher than against Gram-negative bacteria. The minimum bactericidal concentrations (MBCs) of aloe-emodin against the *Staphylococcus* strains were four times the MICs. The maximum non-toxic dose of aloe-emodin to the mouse macrophage RAW 264.7 cell line, as determined by the MTT method, was 100 μg/mL (Figure 2), which was more than the MICs. Thus, aloe-emodin can safely control Gram-positive bacterial infections.

**TABLE 1:** Minimum inhibitory concentrations (μg/mL) of aloe-emodin and other antibiotics against various bacterial species.

	**Phenotypic characteristics**	**Aloe-emodin**		**Oxacillin**	**Levofloxacin**	**Vancomycin**
		**MIC**	**MBC**	**MIC**	**MIC**	**MIC**
*Staphylococcus aureus* M2 CVCC1882 (MSSA)	Drug-sensitive strains	32	128	8	2	0.5
*Staphylococcus aureus* ATCC 33591 (MRSA)	Resistant to methicillin. mecA positive standard strain, pvl gene deletion strain	16	64	512	32	2
*Staphylococcus aureus* ATCC43300 (MRSA)	Resistant to methicillin, mecA positive standard strain, pvl gene deletion strain	32	128	1024	32	4
*Staphylococcus epidermidis* BNCC102555 (MSSE)	Drug-sensitive strains	32	128	4	2	0.5
*Staphylococcus epidermidis* ATCC12228	Drug-sensitive strains	4	16	0.25	0.25	0.25
*Streptococcus pneumoniae* ATCC 49619		16	64	0.5	0.5	0.5
*Escherichia coli* BNCC133264		128	>1024	128	2	4
*Escherichia coli* ATCC 25922		256	>1024	64	4	4
*P. aeruginosa* ATCC 27853		256	>1024	64	8	8
20 strains of *Staphylococcus epidermidis* strains (Clinic isolate)		4–32	32–128	2–16	1–32	0.5–1

*MRSA, methicillin-resistant *Staphylococcus aureus*; MRSE, methicillin-resistant *Staphylococcus epidermidis*; MSSA, methicillin-sensitive *Staphylococcus aureus*; MSSE, methicillin-sensitive *Staphylococcus epidermidis.**

**FIGURE 3:**
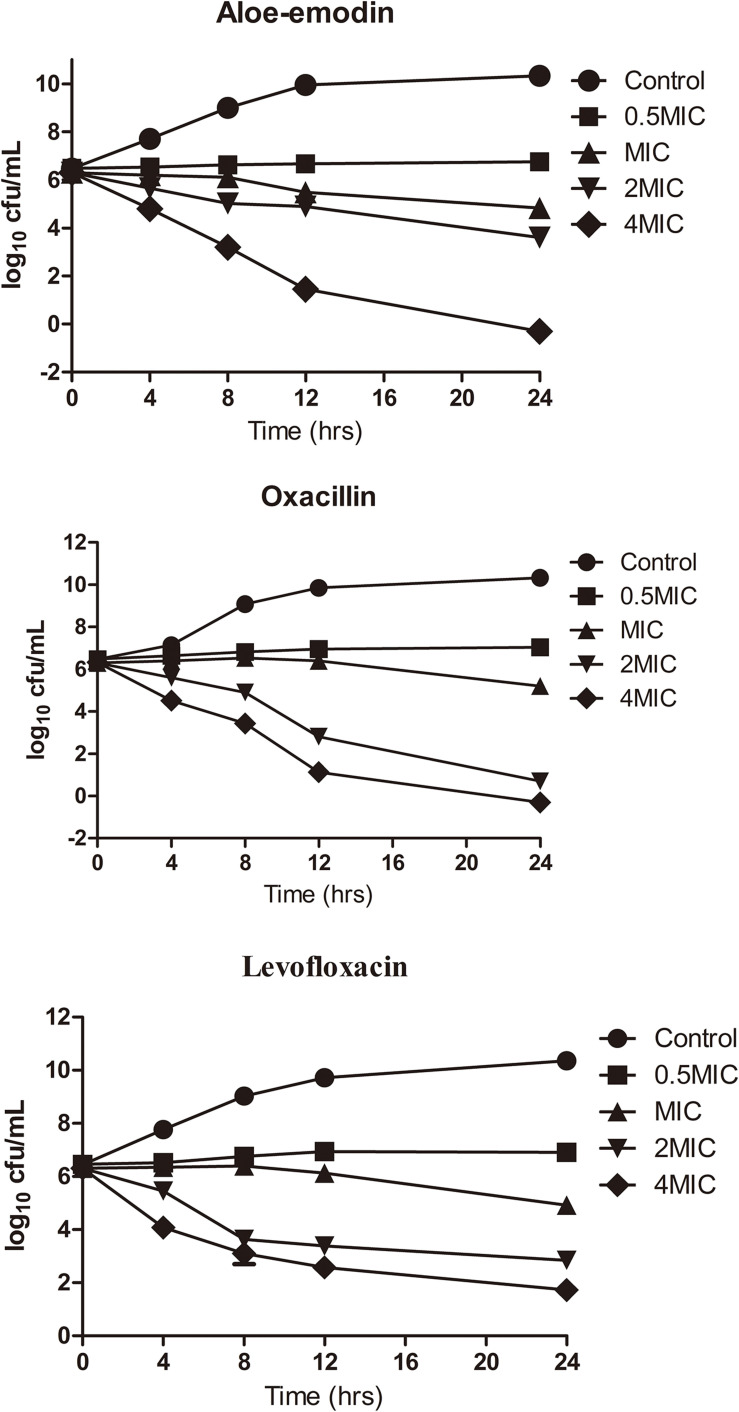
Killing kinetics of aloe-emodin against *Staphylococcus epidermidis* strain BNCC102555. Change in CFU/mL at 0, 4, 8, 12, and 24 h after the addition of 0.5×, 1×, 2×, and 4× the MIC of aloe-emodin, levofloxacin, and oxacilin. A non-treatment group was included as a control. The values plotted are the mean ± S.E.M for three replicates. After 4 h of incubation, the number of live bacteria in the control was significantly higher than in the other groups (*P* < 0.05).

Time-killing assays for cultivable cells were performed to evaluate the killing kinetics of aloe-emodin, oxacillin, and levofloxacin against methicillin-resistant *S. epidermidis* (MRSE) strain BNCC102555, which is resistant to oxacillin. Time-killing curves showed that aloe-emodin (4 × MIC = 128 μg/mL) reduced the initial inoculum from 6.22 ± 0.08 log_10_ colony-forming units (CFU)/mL to 4.79 ± 0.07 log_10_ CFU/mL within 4 h of treatment (Figure 3). Generally, the initial inoculum decreased by 2.13 log_10_ or 1.7 log_10_ following treatment with levofloxacin (4 × MIC = 8 μg/mL) or oxacillin (4 × MIC = 16 μg/mL), respectively. It should be pointed out that *S. epidermidis* isolates are able to enter a dormant state *in vitro* induced by antibiotics, in which the cells are viable but non-cultivable (Carvalhais et al., 2018). In this study, cultivable cells were used to determine the time-killing kinetics. If the viable bacteria count method is used, the bactericidal efficacy may be reduced.

### Bacterial Morphology

**FIGURE 4:**
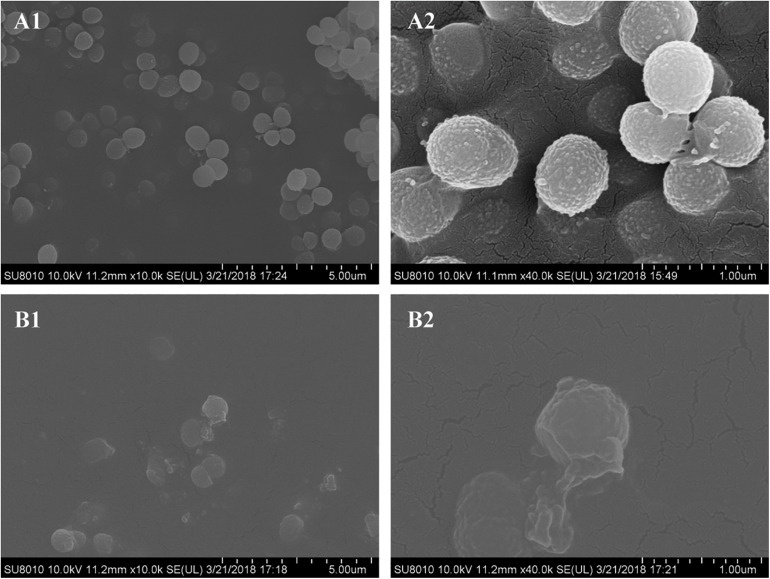
Scanning electron microscopy images of biofilms formed by *Staphylococcus epidermidis* following 12 h of treatment with 16 μg/mL aloe-emodin. Treatment without **(A)** and with aloe-emodin **(B)**. **A2** and **B2** show higher magnifications than **A1** and **B1**, respectively. Scale bars of 5 μm and 1 μm are shown at the bottom right of the panels marked **(A)** and **(B)**, respectively, with each scale bar equally divided into 10 units.

The morphology of *S. epidermidis* cells was examined by scanning electron microscopy (SEM). Untreated cells displayed a spherical shape with smooth and integral cell surfaces (Figures 4A1,A2). Aloe-emodin-treated cells became rough, swollen, and deformed, with typical pitting and disintegration on the cell surface (Figures 4B1,B2).

### Cytoplasmic Membrane Permeability Assay

**FIGURE 5:**
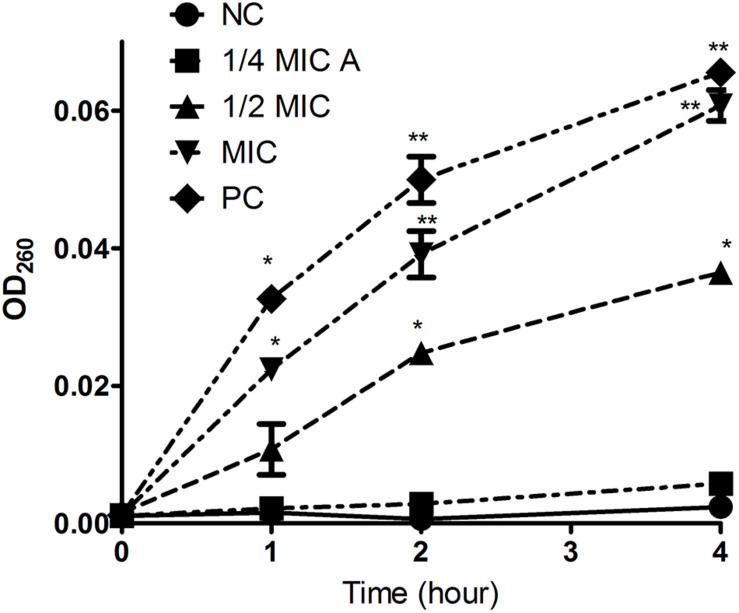
Leakage kinetics of intracellular substances in *Staphylococcus epidermidis*. Aloe-emodin increased cytoplasmic membrane permeability at concentrations of 1/4 MIC, 1/2 MIC, and 1 MIC. Oxacillin (1 MIC) was used as a positive control (PC), and untreated cells were used as a negative control (NC). The values presented are the mean ± S.E.M for three replicates. Single asterisk (*) indicates *P* < 0.05 and double asterisks (**) indicate *P* < 0.01 vs. NC.

Intracellular substances include nucleic acids, proteins, and metabolites with ultraviolet-absorbing properties, and the cell membrane selectively retains these substances. Therefore, the permeability of the cell plasma membrane was measured by quantifying the ultraviolet absorption of the extracellular solution. Aloe-emodin induced the release of 260 nm-absorbing material from bacterial cells in a dosage- and time-dependent manner. As shown in Figure 5, the negative control showed the lowest amount of substance, and the positive control showed the highest amount of substance (PC, oxacillin, 1 × MIC = 4 μg/mL). Within 1 to 4 h, the absorbance values of the aloe-emodin treatment groups gradually increased to a higher level than the negative control group (*P* < 0.01). The amount of leakage detected in the presence of 1 × MIC of aloe-emodin was close to that detected following treatment with oxacillin. These results imply that aloe-emodin and oxacillin increase the cytoplasmic membrane permeability of this strain.

### Quantification of Endogenous Reactive Oxygen Species (ROS)

**FIGURE 6:**
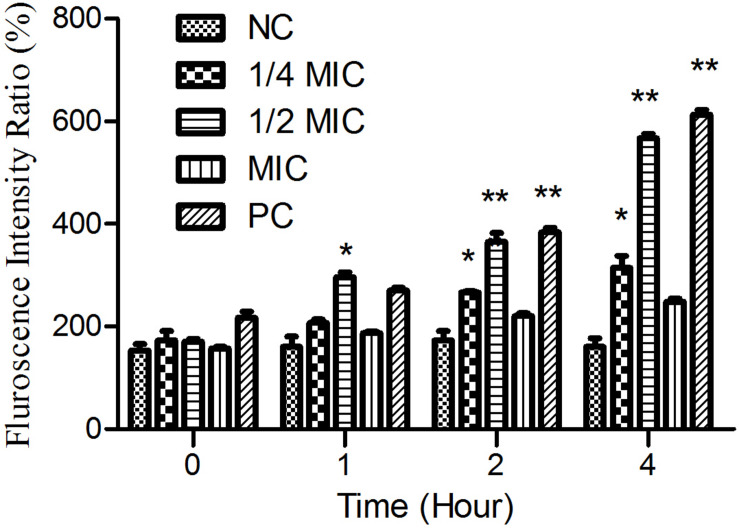
The amount of ROS released from *Staphylococcus epidermidis* exposed to five different treatments over time. Aloe-emodin promotes endogenous ROS production at concentrations of 1/4 MIC, 1/2 MIC, and 1 MIC. Oxacillin (1 MIC) was used as a positive control (PC) and untreated cells were used as a negative control (NC). The statistical analysis of ROS production was compared with the NC. Single asterisk (*) indicates *P* < 0.05 and double asterisks (**) indicate *P* < 0.01 vs. NC.

As shown in Figure 6, the treatment of cells with both aloe-emodin and PC (1/2 MIC of oxacillin) induced the generation of ROS at the 1-h time point, and these differences were significant (*P* < 0.05) compared with the negative control. Treatment with 1/4 and 1/2 MIC of aloe-emodin, resulted in a gradual increase in ROS levels, in a dose- and time-dependent manner. The ROS level following the 1 × MIC treatment was below those detected following the 1/4 and 1/2 MIC treatments; the reason for this may be that bacterial replication was inhibited under the MIC of aloe-emodin.

### Transcriptomic Analysis

**FIGURE 7:**
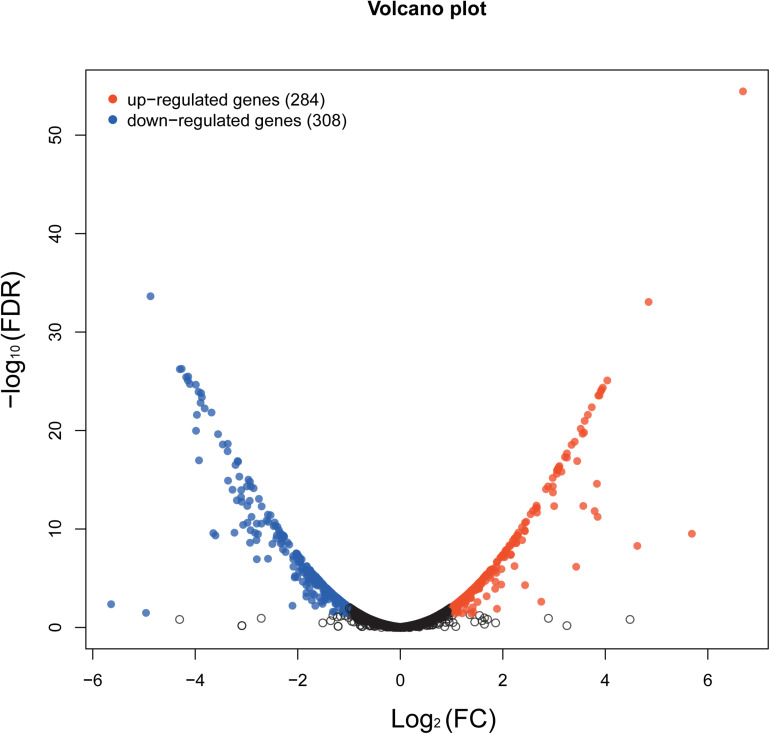
Volcano map showing the overall scatter of differentially expressed genes (DEGs) in *Staphylococcus epidermidis* with or without aloe-emodin treatment. The abscissa indicates the fold-change in gene expression between the two groups (treatment/control). The ordinate indicates the statistical test values for the differences in the fold-change of expression of the transcripts. Each point in the figure represents a specific gene or transcript. Red dots indicate a significantly upregulated gene, blue dots indicate a significantly downregulated gene, and black dots indicate a non-significant change in gene expression. The further the dot is located toward the outside and top of the map, the higher the significant difference in expression.

The whole-transcriptome profile was analyzed by RNA-sequencing (RNA-seq). Transcript levels were compared between and within untreated samples normalized by calculating the reads per kilobase million reads (RPKM). A total of 2,187 genes were analyzed, equivalent to 82% of the *S. epidermidis* genome (total number of genes = 2662). In total, 592 genes were significantly affected by aloe-emodin treatment [*P* < 0.05, Kal’s test with false discovery rates (FDR)], among which 284 genes were upregulated and 308 were downregulated (Supplementary Tables 1, 2). More than 60% of transcripts had RPKM values of up to 250. A volcano map, which integrated both the *P*-value and fold-change of each transcript, was constructed to present the general scattering of the transcripts and filtered the differentially expressed genes (DEGs) following aloe-emodin treatment (Figure 7), Transcriptomic analysis was confirmed to be consistent with the results of real-time PCR (Supplementary Figure 1).

### Gene Ontology (GO) Enrichment and Functional Analysis

**FIGURE 8:**
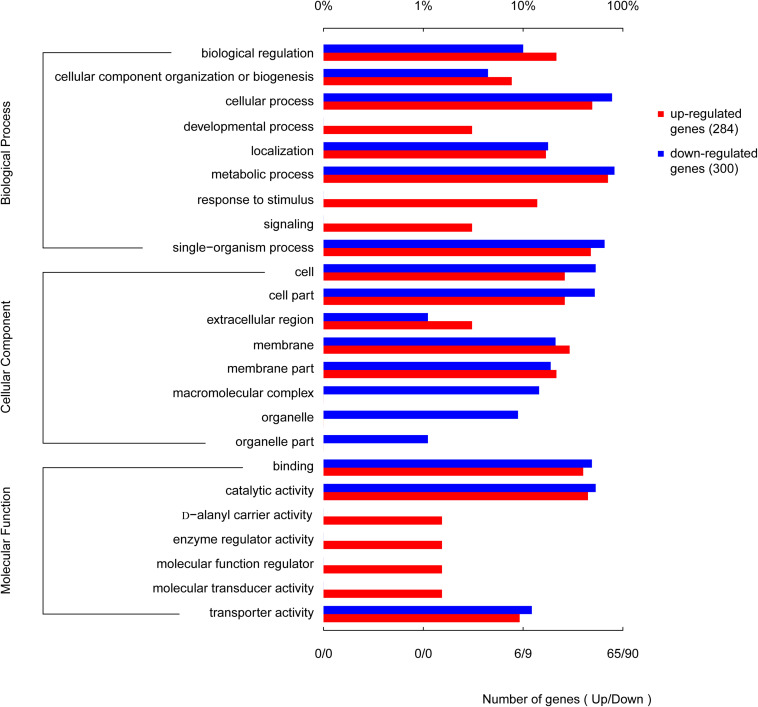
Functional categories of the differentially expressed genes (DEGs), broadly separated into biological processes, cellular components, and molecular function based on Gene Ontology (GO) enrichment analysis. The lower abscissa indicates the number of genes annotated to a certain GO term, while the upper abscissa indicates the percentage of genes annotated to a certain GO term amongst the total number of annotated genes.

When the corrected *P*-values of the FDRs were <0.05, the GO function was considered significantly enriched. In total, 592 genes were mapped to 24 GO terms (Figure 8). The top 10 enriched GO functions included lysine metabolite, diaminopimelate biosynthetic and metabolite, lysine biosyntheses, aspartate-family amino acid metabolite, small-molecule catabolism, interspecies interaction between organisms, pathogenesis, and anion transport. The biological process categories included metabolite (30.2%), cell (25.6%), single-organism (22.6%), localization (6.8%), biological regulation (5.8%), cellular component organization or biogenesis (4.5%), biological regulation (3.5%), signaling (0.5%), and development (0.5%). The molecular function category included catalytic activity (46.6%), binding (42.7%), and transporter activity (10.7%). Processes associated with the production of cells (29.7%), cell parts (29.2%), membranes (17.4%), membrane parts (12.3%), and macromolecular complexes (5.9%) made up a considerable fraction (94.5%) of the cellular components category, along with other important components of organelles (3.7%), organelle parts (1.4%), and extracellular regions (0.45%). Further analysis revealed that genes involved in the development, response to stimuli, enzyme regulator activity, D-alanyl carrier activity, molecular transducer activity, and molecular function regulator activity were all upregulated. Genes involved in the production of macromolecular complexes, organelles, and organelle parts were all downregulated (Figure 8).

### Analysis of Enriched KEGG Pathways

**FIGURE 9:**
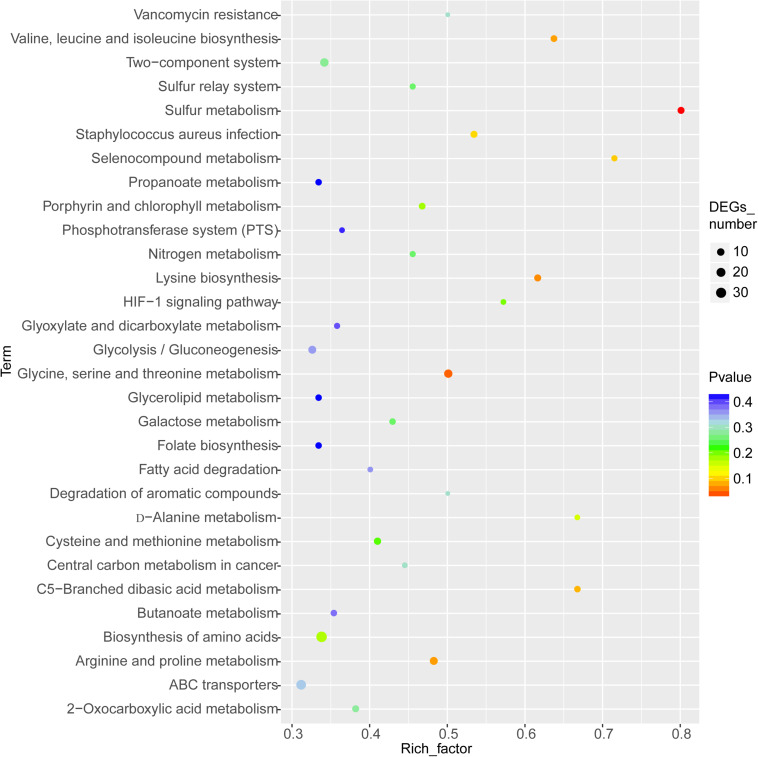
Kyoto Encyclopedia of Genes and Genomes enrichment analysis of the differentially expressed genes (DEGs). The horizontal axis represents the enrichment factor [enriched to a Gene Ontology (GO) term]. The number of DEGs accounts for the ratio of the number of background genes obtained by sequencing. The ordinate indicates the GO term enriched features. The larger the circle, the greater the number of DEGs enriched in this function.

**FIGURE 10:**
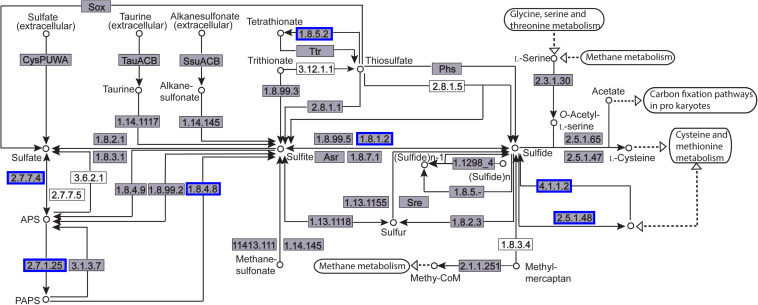
Distribution of the differentially expressed genes (DEGs) in the sulfur metabolism pathway. Compared with the control group, an enzyme with a blue frame is related to a downregulated gene, an enzyme with a red frame is related to an upregulated gene. The number within the frames represents the enzyme code.

**FIGURE 11:**
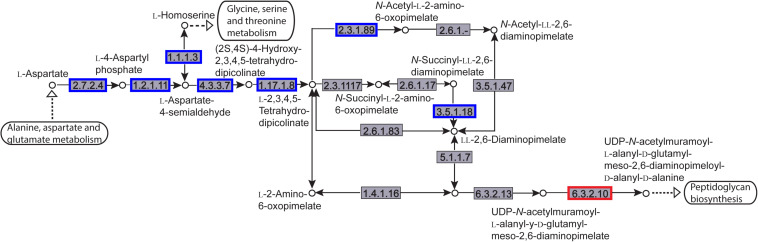
Distribution of the differentially expressed genes (DEGs) in a peptidoglycan biosynthesis pathway. Compared with the control group, an enzyme with a blue frame is related to a downregulated gene, an enzyme with a red frame is related to an upregulated gene. The number within the frames represents the enzyme code.

The enriched KEGG pathways are shown in Figure 9. The sulfur metabolism pathway was the most enriched, and within this pathway, eight DEGs were significantly downregulated, which encoded the following enzymes: the assimilatory sulfite reductase (NADPH) flavoprotein subunit (CPZ21_11540, EC 1.8.1.2), NADPH-dependent assimilatory sulfite reductase hemoprotein subunit (CPZ21_11545, EC 1. 8. 4. 8), cystathionine gamma-synthase (CPZ21_10075, EC 2.5. 1. 48), sulfate adenylyl transferase (sat, EC 2. 7. 7. 4), adenylyl-sulfate kinase (cysC, EC 2. 7. 1. 25), DoxX family protein (CPZ21_08425, EC 4. 4. 1. 2), and phosphoadenylyl-sulfate reductase (CPZ21_11535, EC 1. 8. 5. 2). Interestingly, as shown in Figure 10, most of the affected genes are involved in the reduction of sulfur. The second most enriched KEGG pathway was the lysine biosynthesis pathway, which included aspartate kinase (CPZ21_05270, EC: 2. 7. 2. 4), aspartate-semialdehyde dehydrogenase (CPZ21_05265, EC: 1. 2. 1. 11), homoserine dehydrogenase (CPZ21_05615, EC: 1. 1. 1. 3), 4-hydroxy-tetrahydrodipicolinate synthase (CPZ21_05255, EC: 4. 3. 3. 7), 4-hydroxy-tetrahydrodipicolinate reductase (CPZ21_05260, EC: 1. 17. 1. 8), tetrahydrodipicolinate *N*-acetyltransferase (dapD, EC: 2. 3. 1. 89), succinyl-diaminopimelate desuccinylase (CPZ21_10715, EC: 3. 5. 1. 18), and UDP-*N*-acetylmuramoyl-tripeptide–D-alanyl-D-alanine ligase (CPZ21_02270, EC: 6. 3. 2. 10). The expression of eight genes in the L-lysine biosynthesis pathway of *S. epidermidis* was disrupted (Figure 11).

In addition, aloe-emodin treatment altered the expression of three genes directly related to biofilm formation and cell adhesion, namely those encoding the anti-holin-like proteins, *IrgA* (CPZ21_00595) and *sdrF* (CPZ21_10160, and cellular wall surface anchor protein (CPZ21_11170).

### Predictive Binding Mode

**FIGURE 12:**
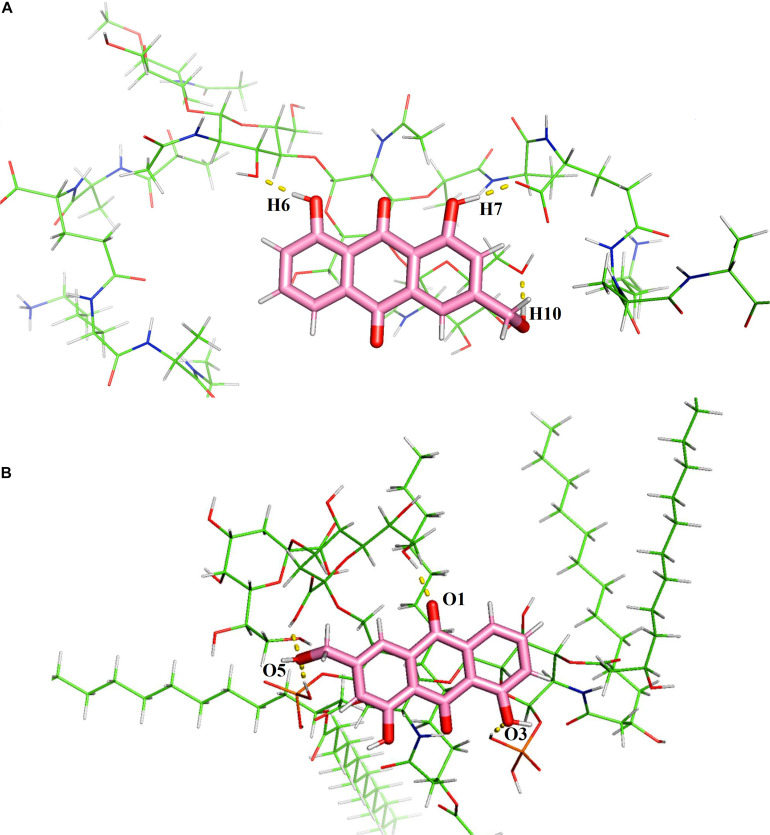
Predicted 3D binding modes of aloe-emodin to the cell membrane. **(A)** The binding of aloe-emodin to peptidoglycan is driven by four hydrogen bonds with H6, H10, O1, and O2. The binding energy is 1425.7789 kJ/mol. **(B)** The binding of aloe-emodin to LPS is driven by three hydrogen bonds H6, H7, and H10. The binding energy is -1945.2531 kJ/mol.

As peptidoglycan and lipopolysaccharide (LPS) are the main components of the cellular envelope of Gram-positive and Gram-negative bacteria, respectively, molecular modeling studies were carried out to predict the binding site and interaction position of aloe-emodin on the cell membrane. The main binding modes are shown in Supplementary Figures 2, 3, and their binding energies are shown in Supplementary Tables 3, 4, respectively. The lowest energy structures of the best conformers for the aloe-emodin–LPS and aloe-emodin–peptidoglycan complexes are presented in Figure 12. The 3D model of aloe-emodin–LPS indicates that the binding of aloe-emodin–LPS is driven by four hydrogen bonds with O1, O3, and O5, and the total energy of aloe-emodin–LPS was −1425.7789 kJ/mol; whereas the binding of aloe-emodin–peptidoglycan was formed by three hydrogen bonds H6, H7, and H10(B), and the total energy of aloe-emodin–peptidoglycan was −1945.2531 kJ/mol. Aloe-emodin is more likely to bind to peptidoglycan and cause membrane perturbation, which indirectly explains why its activity against Gram-positive bacteria is greater than that against Gram-negative bacteria.

## Discussion

The prevalence of antibiotic resistance among *S. epidermidis* isolates is increasing (Haas et al., 2010; Chan et al., 2018; Rossi et al., 2018; Rolta et al., 2020), which highlights the need to identify new anti-bacterial molecules with divergent antibacterial mechanisms. Aloe-emodin has a variety of biological activities (Dong et al., 2020), including inhibiting bacterial biofilm formation (Xiang et al., 2017). The pharmacological properties of aloe-emodin warranted further antibacterial research.

The MICs of aloe-emodin were independent of the resistance levels to other drugs, indicating its unique anti-bacterial mechanism. The MBCs and the results of time-killing assays demonstrated that aloe-emodin has rapid, concentration-dependent, bactericidal activity, as bactericidal antibiotics are defined as those with an MBC to MIC ratio ≤4 (Arce et al., 2011). Emodin is a homolog of aloe-emodin, and the MIC of aloe-emodin was lower than that of emodin against *S. epidermidis* (Beattie et al., 2010). Emodin also exhibited efficacy against *Pseudomonas aeruginosa* (Kohanski et al., 2010).

The cell morphology of *S. epidermidis* was examined using SEM and indicated that treatment with aloe-emodin deformed the outer membrane of the bacteria. Permeability measurements of the cytoplasmic membrane confirmed abnormal membrane function. Molecular simulation confirmed that aloe-emodin targeted the membrane. Based on physicochemical interactions, the development of resistance may be slow.

The activities of some antibacterial drugs are mediated not simply by the initial drug–target interaction, but also by subsequent destruction of cellular homeostasis, especially the generation of lethal ROS within the bacteria (Solis et al., 2016). The transcriptional profile also indicated signs of oxidative stress in the form of increased expression of genes encoding antioxidant proteins. These genes encode NAD(P)H-dependent oxidoreductase, NAD(P)/FAD-dependent oxidoreductase, superoxide dismutase, glutathione peroxidase, and peroxiredoxin, which play a central role in maintaining cellular homeostasis. Oxidative stress may suppress bacterial replication and reduce the production of the extracellular matrix associated with biofilm formation (Motta et al., 2015).

According to transcriptomics analysis, aloe-emodin greatly affected sulfur metabolism in *S. epidermidis*. The metabolism of sulfur involves many physiological and biochemical processes. Sulfur, in its reduced form, is used in the biosynthesis of L-cysteine, L-methionine, and glycopeptides. L-Cysteine is synthesized into biomolecules such as proteins, coenzymes, and L-glutathione. L-Cysteine andL-glutathione are associated with bacterial biofilm formation (Soutourina et al., 2009). Glutathione regulates the cellular redox status and is associated with the synthesis of hydrogen sulfide (H_2_S), which restores intestinal microbiota biofilm formation (Shang et al., 2013). Sulfate assimilation, and the cysteine/methionine biosynthesis pathways in *S. epidermidis*, contribute to biofilm formation (Soutourina et al., 2009). Inactivation of CysI inhibits biofilm formation and adhesion in *Bacillus subtilis* (Fazius et al., 2013; Kobayashi, 2019). CymR is the master regulator of cysteine metabolism and mutation of *cymR* in *S. aureus* leads to a reduced capacity to form biofilms (Otto, 2018). In one study, the biosynthesis and metabolism of L-cysteine, L-cystine, L-glutathione, and L-methionine were suppressed by aloe-emodin, potentially reducing (Wolcott et al., 2010). Additionally, the biosynthesis of L-lysine and endo-diaminopimelate were suppressed, which are the upstream raw materials of peptidoglycan biosynthesis (Mann et al., 2009). Insufficient l-lysine and endo-diaminopimelate may therefore contribute to cell wall defects.

Staphylococci in plankton exhibit a phenotype in terms of metabolism, gene transcription, and protein production, which is different from the phenotype observed during biofilm formation (Lourtet-Hascoet et al., 2018). Planktonic cells and the early stage of biofilm formation may be important therapeutic windows (Schuster et al., 2018). The staphylococcal biofilm matrix consists of polysaccharide intercellular adhesion proteins and extracellular DNA (Lourtet-Hascoet et al., 2018). The *cid* and *lrg* gene products control cell lysis and genomic DNA release during biofilm development (Patti et al., 1994). In the early stages of biofilm formation, *S. aureus* cells passively adsorb onto the material surface and form a monolayer, then microbial surface components recognizing adhesive matrix molecules (MSCRAMM) promote (Lourtet-Hascoet et al., 2018). In a previous study, *sdrF* (CPZ21_10160), which encodes an LPXTG-motif containing protein, was inhibited and found to covalently attach to the bacterial cell surface as an anchor point for adhesion. Suppression of IrgA (CPZ21_00595) reduced extracellular DNA cleavage, release, and biofilm strength.

In conclusion, aloe-emodin has anti-bacterial activity against several clinically representative Gram-positive strains, including some drug-resistant bacterial strains, such as MRSA/MRSE and VRA/VRE. From the biochemical profile, the mode of action may involve binding of the peptidoglycan layer in the cell outer membrane and disruption of cell membrane permeability, eventually causing metabolic disruption and cell death. Further studies are warranted into the biological activity and, in particular, the antibacterial mechanism of aloe-emodin.

## Experimental

### Bacterial Strains and Media

*Staphylococcus aureus*, ATCC 33591, *S. aureus* ATCC43300, *S. epidermidis* ATCC12228, *S. epidermidis* BNCC102555, and *Escherichia coli* BNCC133264, *Escherichia coli*

ATCC 25922 were obtained from Beijing Century Aoke Biotechnology (Beijing, China). *S. aureus* M2 CVCC1882 was obtained from the China Veterinary Institute (Beijing, China) *Streptococcus pneumoniae* ATCC 49619 and *P. aeruginosa* ATCC 27853 were supplied from the China Center of Industrial Culture Collection (CICC) (Beijing, China). Clinical isolates of *S. epidermidis* were collected from cases of bovine mastitis and were identified in our laboratory. Bacterial growth media were all obtained from Beijing Aoboxing Biology Technology Co., Ltd., Beijing, China. All bacterial strains were stored at 4-80°C until use. Bacterial medium and reagents were purchased from Qingdao Hope Bio-Technology Co., Ltd. (Shandong, China). The mouse macrophage cell line, RAW 264.7, was purchased from the American Type Culture Collection (ATCC, Rockville, MD, United States). Dulbecco’s modified Eagle’s medium supplemented with 10% fetal bovine serum was supplied by Gibco (Grand Island, NY, United States).

### Antibacterial Activity Assays

The minimum inhibitory concentrations (MIC) and minimum bactericidal concentrations (MBC) of aloe-emodin (97%; Xi’an Dingjian Bio-Technology Co., Ltd., Xi’an, China) were determined using broth microdilution methods in sterile 96-well microplates, as described by the Clinical and Laboratory Standards Institute (Soutourina et al., 2009), with modifications. Briefly, two-fold serial dilutions of aloe-emodin were prepared in Mueller–Hinton broth (MHB) and were added to 96-well polystyrene plates containing 100 μL of bacterial suspension per well at a concentration of 2 × 10^5^ colony-forming units (CFU)/mL. Columbia medium containing 5% sheep blood was used for growing *S. pneumoniae*. *E. coli* was cultured in lysogeny broth (LB). A control well containing only bacterial inoculum was also included in each plate. Samples were incubated at 37°C for 24 h, and the inhibition of bacterial growth was monitored by measuring the optical density at 600 nm in a microdilution assay. The MIC was defined as the lowest aloe-emodin concentration with no growth at the end of the experiment (Shang et al., 2013). The lowest concentration of aloe-emodin that killed 99.9% of bacteria was defined as the MBC. To determine the MBC, 5 μL aliquots were collected, serially diluted in phosphate-buffered saline (PBS), and plated onto Mueller–Hinton agar plates, in triplicate. Cultivable cells were counted following incubation for 24 h at 37°C. Oxacillin was selected as quality control for *Staphylococcus aureus* ATCC 33591, *Staphylococcus aureus* ATCC43300, Vancomycin was selected as control for the prevalence of vancomycin resistance, and Levofloxacin was selected as positive control for the antibacterial activity assays.

### Time Killing Curves

A 1:200 dilution (1 × 10^6^ CFU/mL) of an overnight culture of *S. epidermidis* (BNCC102555) was inoculated into 20 mL of fresh MHB and cultured aerobically at 37°C until logarithmic phase (optical density at 600 nm = 0.2). Bacterial cells were then collected and diluted to ∼10^6^ CFU/mL with fresh MHB. Dilutions of aloe-emodin at concentrations ranging from 8 μg/mL (1/4 × MIC) to 128 μg/mL (4 × MIC) were added to the bacterial cultures and incubated for 24 h at 37°C with shaking (160 rpm). Aliquots were collected at specified time points and cell counts were performed as above. The rate of killing was determined by plotting CFU/mL against time.

### *In vitro* Cytotoxicity

The mouse macrophage RAW 264.7 cell line was cultured in Dulbecco’s modified Eagle’s medium supplemented with 10% fetal bovine serum and aloe-emodin (1, 10, 20. 50, 100, and 150 μg/mL) to a total volume of 500 μL. After 24 h incubation, culture media containing 3-(4, 5-dimethylthiazol-z-yl)-2, 5-diphenyltetrazolium bromide (MTT) (0.1 mg/mL) was added to each well. Culture media were removed 1 h later, and formazan crystals were dissolved in dimethyl sulfoxide (DMSO) and measured at 550 nm using a microplate reader (BioRad Instruments, United States).

### Detection of Bacterial Morphology by SEM

*S. epidermidis* (BNCC102555), grown in MHB, was incubated with 1/2 × MIC of aloe-emodin in six-well plates containing glass coverslips at 37°C for 8 h. Following incubation, the coverslips were washed in PBS and fixed with 2.5% glutaraldehyde (v/v) for 12 h at 4°C. The coverslips were then dehydrated using an ethanol gradient and coated with a 10-nm layer of gold/palladium. SEM images were observed using a Hitachi S-5000 field emission scanning electron microscope (Hitachi Hi-Technologies Corp., Ltd., Tokyo, Japan). Bacteria cultured in MHB without aloe-emodin were used as a control.

### Cytoplasmic Membrane Permeability

The cytoplasmic membrane permeability was measured by determining the concentration of released UV-absorbing material using UV-VIS spectrophotometry similarly to a previous study (Fazius et al., 2013) with the following modifications. *S. epidermidis* (BNCC102555) was prepared in MHB with shaking at 100 rpm for 4 h at 37°C until log phase. Bacterial cultures were adjusted in saline to 5 × 10^6^CFU/mL, and 1.0 mL was added to 9.0 mL of 2.5 mmol/L sodium HEPES buffer (pH 7.0) supplemented with 100 mmol/L glucose plus 8, 16, and 32 μg/mL (1/4, 1/2, and 1 MIC) of aloe-emodin in individual flasks to achieve a final bacterial concentration of 5 × 10^5^ CFU/mL. Flasks containing the cell suspension without an antibacterial agent were included as a negative control, and those with 4 μg/mL oxacillin were included as a positive control. The bacterial suspension was cultured with shaking at 37°C. The cytoplasmic membrane permeability was determined after 0, 1, 2, and 4 h of incubation. Samples (1.0 mL) were taken at each time point and filtered through a sterile nitrate cellulose membrane (0.22 μm). The OD _260 *nm*_ value of the supernatant was taken and used as an indicator of the extracellular UV-absorbing materials released by the cells. All measurements were conducted in triplicate on a TU-1901 UV/VIS spectrophotometer (Puxi General Instrument Co., Ltd., Beijing, China).

### Detection of ROS

The accumulation of ROS in the cell culture medium was measured using 6-chloramethly-2′,7′-dichlorofluorescein diacetate (CM-DCFDA) (Otto, 2018) (J&K Scientific, Ltd., Chengdu, China) after 0, 1, 2, and 3 h of exposure to aloe-emodin and oxacillin, respectively. The suspension of *S. epidermidis* was centrifuged at 5000 g for 5 min. The cell supernatant and CM-H_2_DCFDA solution were mixed and incubated at 37°C for 30 min in the dark. Then, the ROS in the samples were detected with maximum excitation and emission spectra at 485 nm and 535 nm, respectively, on a fluorescence spectrometer (Hitachi, Japan).

### Transcriptomic Sequencing and Analysis

*Staphylococcus epidermidis* (BNCC102555) in MHB was inoculated without antibiotics (negative control) and with aloe-emodin (16 μg/mL) for 24 h at 37°C. Total RNA was extracted using TRIzol reagent (Invitrogen, Carlsbad, CA, United States), and then treated with DNase I (TaKaRa Bio Inc., Kusatsu, Japan). RNA quality was determined using a Bioanalyser 2100 (Agilent Technologies, Santa Clara, CA, United States; ratio of 23S/16S ≥ 1.2, threshold of RNA integrity number > 5) and quantified using a NanoDrop 2000 spectrophotometer (Thermo Fisher Scientific, Waltham, MA, United States). RNA transcriptome libraries were constructed using a TruSeq RNA sample preparation kit (Illumina, San Diego, CA, United States). rRNA was removed using a Ribo Zero Magnetic kit (G + /G-Bacteria) (Epicenter, Madison, WI, United States) and fragmented in RNA Fragmentation Buffer (Illumina, San Diego, CA, United States). Complementary DNA (cDNA) synthesis, end repair, A-base addition, and ligation of Illumina-indexed adaptors were completed as per the guidelines provided by Illumina Inc. PCR amplifications were carried out using Phusion DNA polymerase (New England Biolabs, Ipswich, MA, United States) with a 15-cycle reaction. Libraries were then size-selected for cDNA target fragments of 200–300 bp using 2% Low Range Ultra Agarose (Bio-Rad, Hercules, CA, United States). After generating the clusters, library sequencing was performed on an Illumina HiSeq platform to produce paired-end reads of 150 bp. Transcriptome sequencing was performed by the Mega Genomics Company (Beijing, China).

The resulting raw paired-end reads were trimmed using SeqPrep^1^ and quality-filtered using Sickle^2^. The reads were aligned against the reference genome of *S. epidermidis* using Rockhopper (Tjaden, 2020). While gene expression was calculated in RPKM. Differential gene expression was analyzed using EdgeR^3^. DEGs were functionally categorized using GO functional enrichment (Gene Ontology, 2020), while KEGG pathway analysis was carried out using GOATOOLS (Kanehisa and Goto, 2000; Tang et al., 2015; Kanehisa et al., 2016; Alrohily et al., 2019). Changes in gene expression of more than two-fold with *P* < 0.05 were regarded as reliable and statistically significant. Kyoto Encyclopedia of Genes and Genomes (KEGG) pathway enrichment analysis was conducted using KOBAS^4^. The corrected KEGG pathways were defined as significantly enriched when *P* < 0.05.

The RNA-seq data were verified by relative quantitative real-time PCR, and the primers used are shown in Supplementary Table 5. Total RNA was isolated from samples using an RNeasy Mini Kit (Qiagen, Hilden, Germany), and 0.5 μg of total RNA was reverse-transcribed with 200 U of Super-Script II (Thermo Fisher, Shanghai, China) using 2.5 μM of random decamer primers (Ambion, Shanghai, China) at 25°C for 10 min and then at 43°C for 1 h. qPCR was performed in triplicate and quantified using SYBR Premix Ex Taq (TaKaRa, Beijing, China) on a 7500 Applied Biosystems Cycler (Analytik Jena, Germany). The cycling parameters were as following: incubation at 95°C for 5 min, followed by 40 cycles at 95°C for 30 s and 60°C for 60 s. Relative quantitation was performed by the 2^–Δ^ ^Δ^ ^CT^ method. The mRNA expression of selected genes was normalized against the internal control (16S rRNA). Primer specificity and the formation of primer dimers were checked by dissociation curves. PCR efficiency was evaluated by calculating the linear regression of the log (fluorescence) to cycle number using Stratagene MXpro software.

### Molecular Modeling

The chemical structure of the LPS, containing lipid A and core sugars, and peptidoglycan were generated by Discovery Studio 2018 (DS2018) (Meroueh et al., 2006; Laguri et al., 2017). The three-dimensional structures of the molecules were optimized by molecular mechanics as implemented using the CHARMm force field with the SMART Minimizer algorithm. The interactions of LPS–aloe-emodin and peptidoglycan–aloe-emodin were analyzed by molecular simulation. For the simulated annealing runs, the Generalized–Born approach was used to treat the solvent implicitly as a dielectric continuum, each conformer was ranked based on the restraint violation, and the final conformers were determined by the resulting energy-minimized structures. All of the computations were performed using DS2018.

### Statistical Analyses

Statistical analyses were conducted using the GraphPad Software package and the Student’s *t*-test was performed. A P value less than 0.05 was considered statistically significant.

## Data Availability Statement

The datasets presented in this study can be found in online repositories. The name of the repository and accession number can be found below: National Center for Biotechnology Information (NCBI) BioProject, https://www.ncbi.nlm.nih.gov/bioproject/, PRJNA673741.

## Author Contributions

XH designed the research, analyzed the data, and wrote the manuscript. TL, YL, HZ, and LW performed the experiments. RB, TL, YJ, WW, and HL revised the manuscript. All authors reviewed the final manuscript.

## Conflict of Interest

LW was employed by company Beijing Huafukang Bioscience Co., Ltd. The remaining authors declare that the research was conducted in the absence of any commercial or financial relationships that could be construed as a potential conflict of interest.

## Publisher’s Note

All claims expressed in this article are solely those of the authors and do not necessarily represent those of their affiliated organizations, or those of the publisher, the editors and the reviewers. Any product that may be evaluated in this article, or claim that may be made by its manufacturer, is not guaranteed or endorsed by the publisher.

## Footnotes

1
https://github.com/jstjohn/SeqPrep

2
https://github.com/najoshi/sickle

3
https://bioconductor.org/packages/release/bioc/html/edgeR.html

4
http://kobas.cbi.pku.edu.cn/home.do
