# Supplementary material for: Antibacterial Activity and Membrane-Targeting Mechanism of Aloe-Emodin Against Staphylococcus epidermidis
Source: Front Microbiol. 2021 Aug 16;12:621866. doi: 10.3389/fmicb.2021.621866 (PMC8415635; doi:10.3389/fmicb.2021.621866)
Supplement: Supplementary file 1 [file Data_Sheet_1.DOCX]

**Antibacterial activity and membrane-targeting mechanism of aloe-emodin against *Staphylococcus epidermidis***

Tao Li ^1, #^, Yan Lu^2, #^, Hua Zhang^2^, Lei Wang^3^, Ross C. Beier^4^, Yajie Jin^1^, Wenjing Wang^1^, Xiaolin Hou^2, *^

1 Shanghai Veterinary Research Institute, CAAS, 518 Ziyue RD, Minhang District, Shanghai, 200241

2. Beijing Key Laboratory of Chinese Veterinary Medicine, National Demonstration Center for Experimental Animal Education, Department of Veterinary Medicine, Beijing University of Agriculture 102206, China

3. Beijing Huafukang Bioscience Co., Ltd., Machikou Town, Changping District, Beijing 102208, China,

4. USDA, Agricultural Research Service, Southern Plains Agricultural Research Center, Food and Feed Safety Research Unit, College Station, TX 77845, USA

#: These authors contributed equally to the study

* Email: hxlsx@163.com


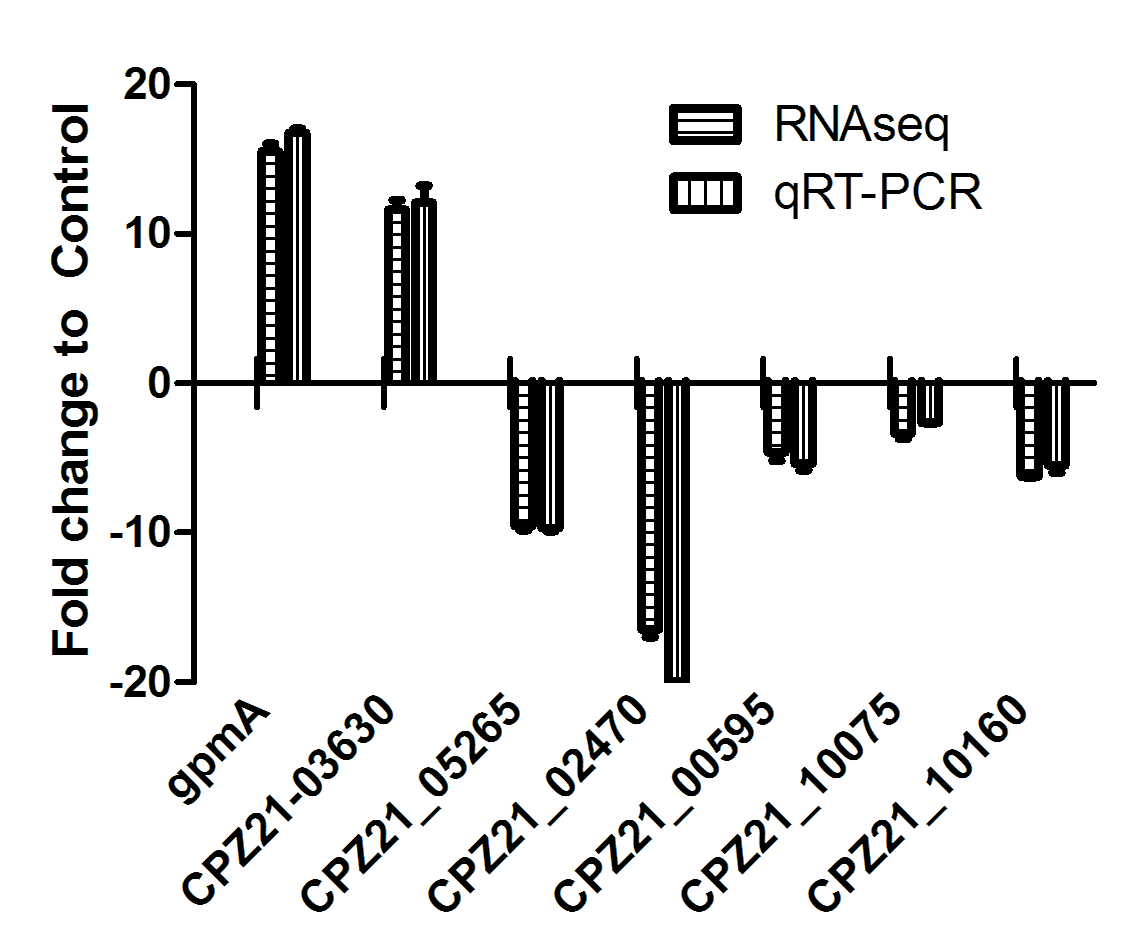


Fig. 1S Validation of RNA sequencing (RNAseq) results by quantitative polymerase chain reaction (qRT-PCR) analysis. the bacterial samples were tested by qRT-PCR analysis. The genes used for validation were selected from the list of genes with significant changes in transcription. Results are shown as the average ± standard deviation of triplicate samples.


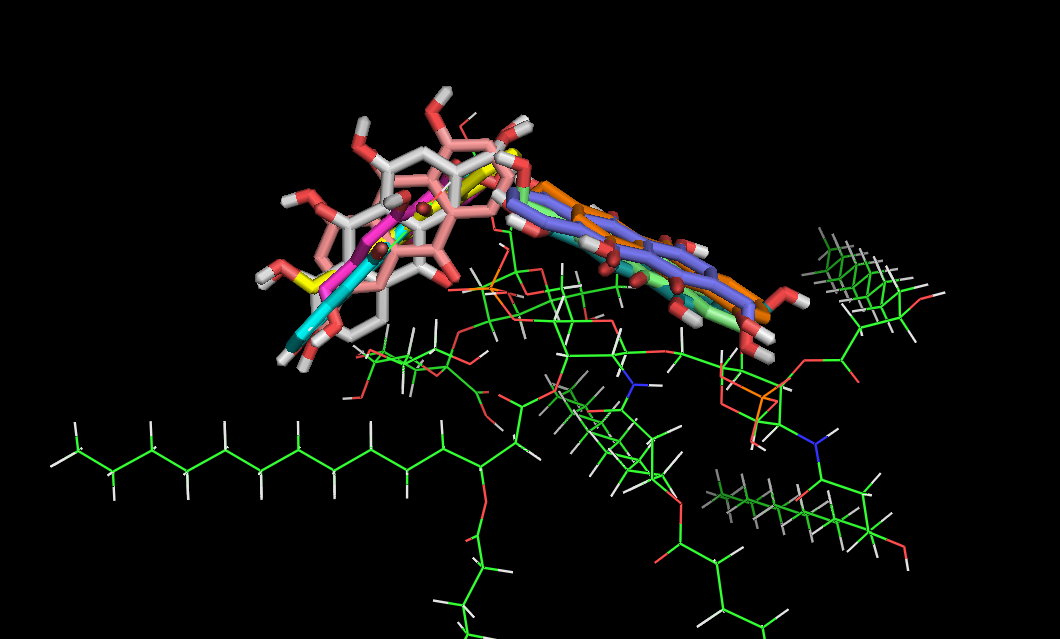


Fig. 2S. 10 binding mode of aloe-emodin on LPS (green line), their total binding energy is shown in table 4S


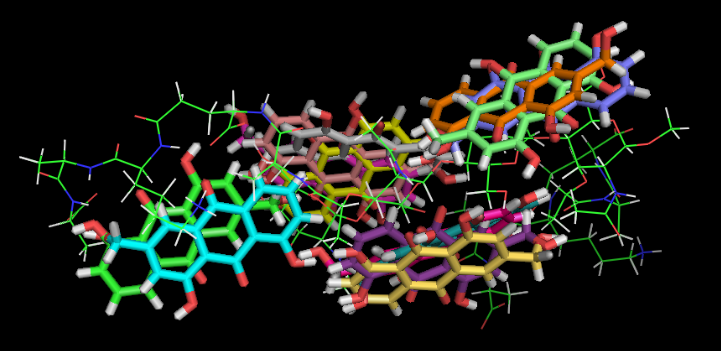


 Fig. 3S. 14 binding mode of aloe-emodin on peptidoglycan (green line).Their total binding energy is shown in table 5S

 Table 1S. Downregulated Genes (Hypothetical proteins were not shown)

| Gene ID | Gene | logFC | pvalue | func |
| --- | --- | --- | --- | --- |
| CPZ21_11230 | gene2245 | -4.87 | 2.11E-37 | peptidase M4 family protein |
| CPZ21_03415 | gene682 | -4.30 | 1.34E-29 | transaldolase |
| CPZ21_10630 | gene2125 | -4.27 | 1.00E-29 | ABC transporter permease |
| CPZ21_05270 | gene1053 | -4.18 | 1.22E-28 | aspartate kinase |
| CPZ21_07435 | gene1486 | -4.14 | 9.06E-29 | argininosuccinate synthase |
| CPZ21_02470 | gene493 | -4.10 | 8.71E-28 | ammonium transporter |
| CPZ21_10640 | gene2127 | -3.99 | 1.13E-27 | DUF5067 domain-containing protein |
| CPZ21_03130 | gene625 | -3.98 | 1.36E-22 | glutamate ABC transporter permease |
| CPZ21_05285 | gene1056 | -3.97 | 2.86E-24 | phosphate-binding protein |
| argH | gene1487 | -3.94 | 8.10E-27 | argininosuccinate lyase |
| glnQ | gene626 | -3.93 | 1.97E-19 | amino acid ABC transporter ATP-binding protein |
| CPZ21_11200 | gene2239 | -3.90 | 1.40E-25 | beta-class phenol-soluble modulin |
| CPZ21_10705 | gene2140 | -3.89 | 1.15E-26 | formate acetyltransferase |
| CPZ21_04840 | gene967 | -3.87 | 3.65E-26 | nucleoside-diphosphate kinase |
| CPZ21_10625 | gene2124 | -3.82 | 5.72E-25 | ABC transporter |
| CPZ21_05255 | gene1051 | -3.56 | 3.33E-22 | 4-hydroxy-tetrahydrodipicolinate synthase |
| CPZ21_05260 | gene1050 | -3.46 | 4.10E-21 | 4-hydroxy-tetrahydrodipicolinate reductase |
| dapD | gene1049 | -3.37 | 2.10E-20 | 2,3,4,5-tetrahydropyridine-2,6-dicarboxylate N-acetyltransferase |
| CPZ21_10695 | gene2138 | -3.37 | 3.39E-21 | lantibiotic ABC transporter ATP-binding protein |
| LeuB | gene476 | -3.36 | 3.16E-17 | 3-isopropylmalate dehydratase large subunit |
| LeuC | gene477 | -3.27 | 3.07E-16 | 3-isopropylmalate dehydrogenase |
| LeuD | gene475 | -3.24 | 1.16E-11 | 3-isopropylmalate dehydratase small subunit |
| CPZ21_05265 | gene1052 | -3.21 | 6.37E-19 | aspartate-semialdehyde dehydrogenase |
| CPZ21_02375 | gene474 | -3.19 | 3.95E-15 | threonine dehydratase |
| CPZ21_11205 | gene2240 | -3.17 | 2.38E-19 | acetoin reductase |
| CPZ21_05245 | gene1048 | -3.14 | 1.17E-17 | amidohydrolase |
| CPZ21_10660 | gene2131 | -3.10 | 3.43E-16 | PF10100 family protein |
| CPZ21_05320 | gene1063 | -3.09 | 5.72E-15 | IS256 family transposase, partial |
| CPZ21_08740 | gene1747 | -3.07 | 1.70E-12 | transposase for insertion sequence element IS256 in transposon |
| CPZ21_11215 | gene2242 | -3.00 | 1.35E-16 | DUF1672 domain-containing protein |
| CPZ21_09855 | gene1970 | -2.98 | 1.58E-14 | IS256 family transposase, partial |
| CPZ21_09850 | gene1969 | -2.98 | 9.73E-13 | IS256 family transposase, partial |
| esaA | gene2258 | -2.96 | 2.46E-17 | type VII secretion protein EsaA |
| CPZ21_02395 | gene478 | -2.94 | 4.50E-15 | 2-isopropylmalate synthase |
| pflA | gene2141 | -2.93 | 4.19E-17 | pyruvate formate-lyase-activating enzyme |
| CPZ21_05540 | gene1107 | -2.93 | 1.49E-10 | transposase, mutator-like family protein, partial |
| CPZ21_05535 | gene1106 | -2.92 | 6.24E-12 | IS256 family transposase, partial |
| CPZ21_11560 | gene2311 | -2.91 | 1.33E-16 | sulfite exporter TauE/SafE family protein |
| sat | gene2312 | -2.86 | 2.10E-16 | sulfate adenylyltransferase |
| CPZ21_10535 | gene2106 | -2.83 | 1.25E-11 | transposase |
| pstC | gene1057 | -2.80 | 1.23E-12 | phosphate ABC transporter permease subunit PstC |
| lacB | gene342 | -2.80 | 8.97E-09 | galactose-6-phosphate isomerase subunit LacB |
| CPZ21_05315 | gene1062 | -2.78 | 1.72E-11 | IS256 transposase, partial |
| CPZ21_10700 | gene2139 | -2.76 | 2.76E-15 | ABC transporter permease |
| pstA | gene1058 | -2.71 | 1.25E-12 | phosphate ABC transporter, permease protein PstA |
| CPZ21_10620 | gene2123 | -2.70 | 1.80E-14 | ABC transporter, ATP-binding protein |
| CPZ21_02725 | gene544 | -2.58 | 1.36E-13 | nitric oxide synthase |
| CPZ21_11235 | gene2246 | -2.58 | 7.93E-09 | ArgR family transcriptional regulator |
| CPZ21_02400 | gene479 | -2.58 | 7.79E-13 | ketol-acid reductoisomerase |
| CPZ21_00595 | gene118 | -2.53 | 1.55E-13 | antiholin-like protein IrgA |
| CPZ21_05300 | gene1059 | -2.50 | 2.07E-10 | phosphate ABC transporter ATP-binding protein |
| cysC | gene2313 | -2.45 | 9.24E-13 | adenylyl-sulfate kinase |
| CPZ21_02475 | gene494 | -2.44 | 2.00E-12 | LacI family transcriptional regulator |
| CPZ21_06250 | gene1249 | -2.42 | 5.23E-11 | uracil permease |
| CPZ21_11285 | gene2256 | -2.41 | 2.31E-11 | CHAP domain-containing protein |
| cobA | gene2309 | -2.40 | 2.66E-12 | uroporphyrinogen-III C-methyltransferase |
| CPZ21_11555 | gene2310 | -2.37 | 6.17E-12 | precorrin-2 dehydrogenase |
| essA | gene2259 | -2.35 | 1.25E-11 | type VII secretion protein EssA |
| CPZ21_01700 | gene339 | -2.33 | 1.85E-10 | NAD-dependent protein deacylase |
| CPZ21_10215 | gene2042 | -2.31 | 4.45E-11 | RND transporter |
| CPZ21_10210 | gene2041 | -2.31 | 1.23E-10 | ABC transporter ATP-binding protein |
| CPZ21_00200 | gene39 | -2.29 | 7.49E-10 | ribose transporter RbsU |
| CPZ21_10160 | gene2031 | -2.27 | 3.26E-11 | sdrF/YSIRK signal domain/LPXTG anchor domain surface protein |
| CPZ21_07525 | gene1504 | -2.27 | 3.21E-11 | sodiumproton antiporter |
| CPZ21_10085 | gene2016 | -2.24 | 1.46E-09 | bifunctional homocysteine S-methyltransferase/methylenetetrahydrofolate reductase |
| CPZ21_10205 | gene2040 | -2.20 | 1.40E-10 | ABC transporter permease |
| CPZ21_09800 | gene1959 | -2.17 | 2.43E-10 | NADH dehydrogenase subunit 5 |
| CPZ21_01730 | gene345 | -2.10 | 0.001483392 | lactose-specific phosphotransferase enzyme IIA component |
| CPZ21_09745 | gene1948 | -2.09 | 3.99E-09 | methionine ABC transporter ATP-binding protein |
| CPZ21_04180 | gene835 | -2.09 | 8.82E-09 | allophanate hydrolase subunit 1 |
| CPZ21_10080 | gene2015 | -2.06 | 8.97E-07 | PLP-dependent transferase |
| lacD | gene344 | -2.05 | 6.16E-07 | tagatose-bisphosphate aldolase |
| nrdG | gene2316 | -2.02 | 4.39E-09 | anaerobic ribonucleoside-triphosphate reductase activating protein |
| CPZ21_00855 | gene170 | -2.01 | 2.24E-09 | PTS sucrose transporter subunit IIBC |
| CPZ21_11305 | gene2260 | -2.01 | 8.21E-07 | type VII secretion protein EsaB |
| CPZ21_11580 | gene2315 | -2.00 | 3.47E-09 | anaerobic ribonucleoside-triphosphate reductase |
| CPZ21_05240 | gene1047 | -1.99 | 1.29E-08 | alanine racemase |
| essB | gene2261 | -1.98 | 1.93E-08 | type VII secretion protein EssB |
| CPZ21_10670 | gene2133 | -1.97 | 1.90E-07 | ABC transporter permease |
| galU | gene86 | -1.96 | 6.80E-09 | UTP--glucose-1-phosphate uridylyltransferase |
| CPZ21_05575 | gene1114 | -1.93 | 8.35E-08 | 30S ribosomal protein S14 |
| CPZ21_08655 | gene1730 | -1.92 | 9.36E-09 | peptidase M23 |
| CPZ21_09740 | gene1947 | -1.90 | 7.11E-08 | ABC transporter permease |
| CPZ21_05615 | gene1122 | -1.90 | 2.45E-07 | homoserine dehydrogenase |
| gap | gene765 | -1.85 | 1.68E-07 | aldehyde dehydrogenase |
| CPZ21_05425 | gene1084 | -1.83 | 0.000130046 | 4-oxalocrotonate tautomerase |
| CPZ21_09995 | gene1998 | -1.83 | 6.42E-06 | histidine phosphatase family protein |
| CPZ21_11545 | gene2308 | -1.83 | 4.90E-08 | NADPH-dependent assimilatory sulfite reductase hemoprotein subunit |
| CPZ21_00590 | gene117 | -1.82 | 4.89E-08 | antiholin-like protein LrgB |
| CPZ21_10150 | gene2029 | -1.82 | 5.83E-07 | glycosyl transferase family 1 |
| nikA | gene2132 | -1.81 | 2.76E-07 | nickel ABC transporter, nickel/metallophore periplasmic binding protein |
| CPZ21_02625 | gene524 | -1.81 | 5.17E-07 | DUF1700 domain-containing protein |
| CPZ21_09390 | gene1877 | -1.79 | 1.38E-07 | NupC/NupG family nucleoside CNT transporter |
| CPZ21_07520 | gene1503 | -1.78 | 1.27E-07 | PaaI family thioesterase |
| CPZ21_01720 | gene343 | -1.77 | 1.30E-05 | tagatose-6-phosphate kinase |
| phoU | gene1060 | -1.77 | 1.63E-06 | phosphate transport system regulatory protein PhoU |
| CPZ21_09795 | gene1958 | -1.77 | 1.27E-07 | DUF2309 domain-containing protein |
| CPZ21_05500 | gene1099 | -1.76 | 1.33E-07 | BCCT family transporter |
| CPZ21_11535 | gene2306 | -1.73 | 3.63E-07 | phosphoadenylyl-sulfate reductase |
| CPZ21_00420 | gene83 | -1.73 | 0.000352487 | GntR family transcriptional regulator |
| CPZ21_01670 | gene333 | -1.72 | 5.68E-07 | energy-coupling factor transporter ATPase |
| lacA | gene341 | -1.71 | 5.50E-05 | galactose-6-phosphate isomerase subunit LacA |
| CPZ21_04535 | gene906 | -1.70 | 4.15E-07 | aminomethyl-transferring glycine dehydrogenase |
| CPZ21_09735 | gene1946 | -1.69 | 1.11E-06 | methionine ABC transporter substrate-binding protein |
| CPZ21_07445 | gene1488 | -1.68 | 7.86E-05 | glycerophosphodiester phosphodiesterase |
| CPZ21_08240 | gene1647 | -1.66 | 4.58E-06 | GrpB family protein |
| CPZ21_05080 | gene1015 | -1.66 | 0.001507801 | ABC transporter ATP-binding protein |
| CPZ21_11290 | gene2257 | -1.65 | 6.39E-07 | WXG100 family type VII secretion target |
| CPZ21_03485 | gene696 | -1.65 | 1.50E-06 | transcriptional regulator |
| glnA | gene1139 | -1.63 | 8.85E-07 | glutamine synthetase |
| CPZ21_00815 | gene162 | -1.63 | 1.01E-06 | transcriptional regulator |
| CPZ21_05275 | gene1054 | -1.60 | 1.41E-06 | ABC transporter ATP-binding protein |
| CPZ21_05610 | gene1121 | -1.60 | 4.64E-06 | threonine synthase |
| CPZ21_11540 | gene2307 | -1.59 | 1.71E-06 | assimilatory sulfite reductase (NADPH) flavoprotein subunit |
| CPZ21_05705 | gene1140 | -1.59 | 1.57E-06 | MerR family transcriptional regulator |
| fba | gene411 | -1.57 | 2.07E-06 | fructose-bisphosphate aldolase |
| CPZ21_01345 | gene268 | -1.56 | 3.70E-06 | urease subunit beta |
| CPZ21_10075 | gene2014 | -1.54 | 0.00053388 | cystathionine gamma-synthase |
| CPZ21_03715 | gene742 | -1.53 | 3.76E-06 | acetate kinase |
| ureC | gene267 | -1.50 | 6.07E-06 | urease subunit alpha |
| CPZ21_10685 | gene2136 | -1.50 | 2.63E-05 | ABC transporter ATP-binding protein |
| gcvT | gene905 | -1.50 | 7.04E-06 | glycine cleavage system protein T |
| metE | gene2017 | -1.48 | 1.35E-05 | 5-methyltetrahydropteroyltriglutamate--homocysteine S-methyltransferase |
| pgsC | gene35 | -1.48 | 0.000356942 | poly-gamma-glutamate biosynthesis protein PgsC |
| CPZ21_00365 | gene72 | -1.48 | 2.70E-05 | MFS transporter |
| CPZ21_10715 | gene2142 | -1.48 | 5.26E-05 | succinyl-diaminopimelate desuccinylase |
| CPZ21_05855 | gene1170 | -1.47 | 9.91E-06 | insulinase family protein |
| CPZ21_05505 | gene1100 | -1.45 | 5.49E-05 | large conductance mechanosensitive channel protein MscL |
| essC | gene2262 | -1.45 | 1.43E-05 | type VII secretion protein EssC |
| CPZ21_10595 | gene2118 | -1.45 | 1.30E-05 | ABC transporter substrate-binding protein |
| CPZ21_03805 | gene760 | -1.44 | 1.47E-05 | sensor histidine kinase |
| ccpA | gene721 | -1.44 | 1.55E-05 | catabolite control protein A |
| CPZ21_0070 | gene13 | -1.43 | 0.0001587 | iron transporter FeoA |
| CPZ21_06280 | gene1255 | -1.43 | 1.53E-05 | isoleucine--tRNA ligase |
| CPZ21_06480 | gene1295 | -1.42 | 1.93E-05 | phenylalanine--tRNA ligase subunit alpha |
| CPZ21_10675 | gene2134 | -1.42 | 7.91E-05 | ABC transporter permease |
| CPZ21_05850 | gene1169 | -1.41 | 2.27E-05 | insulinase family protein |
| brnQ | gene1035 | -1.41 | 2.24E-05 | branched-chain amino acid transport system II carrier protein |
| CPZ21_02720 | gene543 | -1.41 | 2.29E-05 | chorismate mutase |
| CPZ21_00205 | gene40 | -1.40 | 0.000276687 | D-ribose pyranase |
| rplU | gene796 | -1.39 | 2.39E-05 | 50S ribosomal protein L21 |
| CPZ21_10680 | gene2135 | -1.39 | 8.32E-05 | ABC transporter ATP-binding protein |
| CPZ21_01335 | gene266 | -1.38 | 3.58E-05 | urease accessory protein UreE |
| CPZ21_06055 | gene1210 | -1.37 | 8.87E-05 | ribosome maturation factor RimM |
| CPZ21_01675 | gene334 | -1.36 | 6.02E-05 | energy-coupling factor transporter ATPase |
| CPZ21_08060 | gene1611 | -1.36 | 3.65E-05 | transcriptional regulator |
| pgsB | gene34 | -1.36 | 8.00E-05 | poly-gamma-glutamate synthase PgsB |
| CPZ21_08725 | gene1744 | -1.35 | 4.90E-05 | nucleoside permease |
| scpB | gene947 | -1.34 | 6.35E-05 | SMC-Scp complex subunit ScpB |
| CPZ21_10730 | gene2145 | -1.33 | 5.49E-05 | 3-keto-5-aminohexanoate cleavage protein |
| CPZ21_03210 | gene641 | -1.33 | 5.43E-05 | YlbF/YmcA family competence regulator |
| CPZ21_04540 | gene907 | -1.32 | 6.50E-05 | glycine dehydrogenase subunit 2 |
| glpT | gene82 | -1.32 | 0.000180776 | glycerol-3-phosphate transporter |
| CPZ21_09210 | gene1841 | -1.32 | 0.008018689 | 2-C-methyl-D-erythritol 4-phosphate cytidylyltransferase |
| CPZ21_00135 | gene26 | -1.29 | 0.000105567 | PTS glucose EIICBA component |
| CPZ21_11740 | gene2147 | -1.29 | 9.86E-05 | thioesterase |
| CPZ21_03275 | gene654 | -1.28 | 0.000111857 | uroporphyrinogen decarboxylase |
| CPZ21_03555 | gene710 | -1.28 | 0.000109624 | tRNA (guanosine(46)-N7)-methyltransferase TrmB |
| CPZ21_11590 | gene2317 | -1.28 | 0.000113092 | C4-dicarboxylate ABC transporter |
| rbsK | gene41 | -1.27 | 0.000230826 | ribokinase |
| ilvB | gene481 | -1.27 | 0.000358911 | acetolactate synthase, large subunit, biosynthetic type |
| CPZ21_07755 | gene1550 | -1.27 | 0.000851414 | GNAT family N-acetyltransferase |
| CPZ21_01350 | gene269 | -1.26 | 0.000157521 | urease subunit gamma |
| CPZ21_00835 | gene166 | -1.26 | 0.000142618 | DUF4889 domain-containing protein |
| CPZ21_00775 | gene154 | -1.25 | 0.00015226 | nitrate reductase subunit alpha |
| CPZ21_01695 | gene338 | -1.24 | 0.000155533 | 30S ribosomal protein S9 |
| CPZ21_06015 | gene1202 | -1.24 | 0.000165077 | succinyl-CoA ligase subunit beta |
| CPZ21_09750 | gene1949 | -1.24 | 0.000173673 | cystathionine gamma-synthase |
| CPZ21_10615 | gene2122 | -1.24 | 0.000193197 | diacetyl reductase ((S)-acetoin forming) |
| CPZ21_03605 | gene720 | -1.23 | 0.000199845 | bifunctional 3-deoxy-7-phosphoheptulonate synthase/chorismate mutase |
| CPZ21_00605 | gene120 | -1.23 | 0.000245662 | sensor histidine kinase |
| CPZ21_10600 | gene2119 | -1.23 | 0.000602273 | FADprotein FMN transferase |
| CPZ21_04685 | gene936 | -1.23 | 0.000194056 | transcriptional regulator, effector binding domain protein |
| CPZ21_10745 | gene2148 | -1.23 | 0.000198851 | glycine/betaine ABC transporter ATP-binding protein |
| CPZ21_06565 | gene1312 | -1.23 | 0.000196025 | protoheme IX farnesyltransferase |
| CPZ21_01320 | gene263 | -1.22 | 0.000221848 | urease accessory protein UreD |
| CPZ21_01690 | gene337 | -1.22 | 0.000216338 | 50S ribosomal protein L13 |
| CPZ21_10735 | gene2146 | -1.21 | 0.000226116 | 3-hydroxybutyryl-CoA dehydrogenase |
| CPZ21_11600 | gene2319 | -1.21 | 0.00025526 | BCCT family transporter |
| CPZ21_03550 | gene709 | -1.20 | 0.00025383 | phosphotransferase enzyme family |
| CPZ21_04290 | gene857 | -1.20 | 0.000330927 | coproporphyrinogen III oxidase |
| CPZ21_10095 | gene2018 | -1.20 | 0.000564323 | arylformamidase |
| narJ | gene156 | -1.20 | 0.000296448 | nitrate reductase molybdenum cofactor assembly chaperone |
| CPZ21_06885 | gene1376 | -1.20 | 0.000268603 | mannosyl-glycoprotein endo-beta-N-acetylglucosamidase |
| feoB | gene14 | -1.19 | 0.000374179 | ferrous iron transport protein B |
| CPZ21_06010 | gene1201 | -1.19 | 0.000309048 | succinyl-CoA ligase subunit alpha |
| CPZ21_05170 | gene1033 | -1.18 | 0.000361178 | ATPase family associated with various cellular activities (AAA) |
| ureG | gene264 | -1.18 | 0.000376016 | urease accessory protein UreG |
| CPZ21_05845 | gene1168 | -1.17 | 0.000391252 | 3-oxoacyl-ACP reductase |
| CPZ21_07550 | gene1509 | -1.17 | 0.00050506 | NAD(P)/FAD-dependent oxidoreductase |
| CPZ21_05175 | gene1034 | -1.17 | 0.000399153 | VWA domain-containing protein |
| CPZ21_09285 | gene1856 | -1.16 | 0.000406923 | 50S ribosomal protein L7/L12 |
| CPZ21_08690 | gene1737 | -1.16 | 0.000444359 | 3-beta hydroxysteroid dehydrogenase |
| CPZ21_01070 | gene213 | -1.15 | 0.000605319 | DUF805 domain-containing protein |
| narH | gene155 | -1.15 | 0.000451082 | nitrate reductase subunit beta |
| CPZ21_01330 | gene265 | -1.15 | 0.000486432 | urease accessory protein UreF |
| CPZ21_04185 | gene836 | -1.15 | 0.000780007 | allophanate hydrolase |
| CPZ21_08920 | gene1783 | -1.15 | 0.000857118 | endonuclease III |
| CPZ21_06475 | gene1294 | -1.15 | 0.000477179 | phenylalanine--tRNA ligase subunit beta |
| CPZ21_09755 | gene1950 | -1.13 | 0.00066749 | cysteine synthase family protein |
| pgk | gene1609 | -1.13 | 0.00060202 | phosphoglycerate kinase |
| CPZ21_02205 | gene440 | -1.12 | 0.000628036 | transglycosylase SceD |
| betB | gene2321 | -1.11 | 0.000707528 | betaine-aldehyde dehydrogenase |
| CPZ21_03950 | gene789 | -1.11 | 0.00447775 | prepilin peptidase |
| CPZ21_08385 | gene1676 | -1.11 | 0.001355543 | anthranilate synthase component I family protein |
| xylB | gene42 | -1.10 | 0.001252147 | xylulokinase |
| CPZ21_08960 | gene1791 | -1.10 | 0.003016448 | 1-(5-phosphoribosyl)-5-amino-4-imidazole-carboxylate carboxylase |
| CPZ21_11750 | gene2149 | -1.10 | 0.000904447 | ABC transporter permease |
| CPZ21_08390 | gene1677 | -1.09 | 0.001797631 | aminodeoxychorismate/anthranilate synthase component II |
| CPZ21_03955 | gene790 | -1.09 | 0.004530252 | JAB domain-containing protein |
| CPZ21_09820 | gene1963 | -1.08 | 0.001505753 | GTP-binding protein |
| CPZ21_10995 | gene2198 | -1.08 | 0.004965353 | histidinol dehydrogenase |
| gap | gene1610 | -1.07 | 0.001032023 | aldehyde dehydrogenase |
| rpsR | gene2004 | -1.07 | 0.00117384 | ribosomal protein S18 |
| CPZ21_00185 | gene36 | -1.06 | 0.002522885 | capsule biosynthesis protein CapA |
| CPZ21_00810 | gene161 | -1.06 | 0.001243001 | NarK/NasA family nitrate transporter |
| CPZ21_03900 | gene779 | -1.06 | 0.001233311 | glutamyl-tRNA reductase |
| CPZ21_08380 | gene1675 | -1.06 | 0.003968895 | aminodeoxychorismate lyase |
| trmL | gene628 | -1.05 | 0.001843376 | tRNA (uridine(34) /cytosine(34)/5-carboxymethylaminomethyluridine(34)  -2'-O)-methyltransferase TrmL |
| CPZ21_10870 | gene2173 | -1.04 | 0.001703539 | sugar porter family MFS transporter |
| CPZ21_03720 | gene743 | -1.04 | 0.001602309 | universal stress family protein |
| CPZ21_10000 | gene1999 | -1.04 | 0.01208529 | LysE family translocator |
| CPZ21_00160 | gene31 | -1.03 | 0.007417872 | YitT family protein |
| CPZ21_05890 | gene1177 | -1.03 | 0.001919953 | tRNA pseudouridine(55) synthase TruB |
| CPZ21_08425 | gene1684 | -1.03 | 0.004785975 | DoxX family protein |
| CPZ21_03875 | gene774 | -1.03 | 0.00205723 | NUDIX domain-containing protein |
| CPZ21_08645 | gene1728 | -1.03 | 0.001900996 | AraC family transcriptional regulator |
| sucB | gene1027 | -1.03 | 0.001791963 | dihydrolipoyllysine-residue succinyltransferase component of 2-oxoglutarate dehydrogenase complex |
| CPZ21_06255 | gene1250 | -1.03 | 0.001879731 | bifunctional pyrimidine operon transcriptional regulator/uracil phosphoribosyltransferase |
| CPZ21_07335 | gene1466 | -1.03 | 0.003940772 | ABC transporter permease |
| CPZ21_08640 | gene1727 | -1.02 | 0.004805746 | accessory regulator family |
| CPZ21_03615 | gene722 | -1.02 | 0.002085934 | formate--tetrahydrofolate ligase |
| CPZ21_06270 | gene1253 | -1.02 | 0.010060558 | CHAP domain-containing protein |
| hemH | gene655 | -1.01 | 0.00202758 | ferrochelatase |
| CPZ21_09260 | gene1851 | -1.01 | 0.002103506 | ribosomal protein S12 |
| CPZ21_06050 | gene1209 | -1.00 | 0.002704137 | tRNA (guanosine(37)-N1)-methyltransferase TrmD |

Table 2S. Up-regulated Genes (hypothetical proteins were not shown)

| Gene ID | Gene | logFC | pvalue | func |
| --- | --- | --- | --- | --- |
| CPZ21_09075 | gene1814 | 6.69 | 1.64E-58 | protein VraX |
| CPZ21_00820 | gene163 | 4.84 | 1.20E-36 | nitroreductase family protein |
| CPZ21_06385 | gene1276 | 4.62 | 3.34E-10 | beta-class phenol-soluble modulin |
| CPZ21_06180 | gene1235 | 4.04 | 3.33E-28 | NINE protein |
| arcA | gene2247 | 3.95 | 2.43E-27 | arginine deiminase |
| gpmA | gene135 | 3.93 | 4.37E-27 | phosphoglycerate mutase |
| CPZ21_03630 | gene725 | 3.91 | 6.91E-27 | serine protease |
| CPZ21_00050 | gene9 | 3.89 | 2.23E-26 | Cu(2+)-exporting ATPase |
| argF | gene2248 | 3.86 | 2.30E-26 | ornithine carbamoyltransferase |
| CPZ21_06390 | gene1277 | 3.85 | 2.35E-13 | beta-class phenol-soluble modulin |
| CPZ21_06380 | gene1275 | 3.79 | 5.63E-14 | beta-class phenol-soluble modulin |
| CPZ21_01985 | gene396 | 3.74 | 4.17E-25 | DNA starvation/stationary phase protection protein |
| CPZ21_02865 | gene572 | 3.66 | 2.93E-24 | sensor histidine kinase |
| arcD | gene2249 | 3.60 | 1.25E-23 | arginine-ornithine antiporter |
| CPZ21_11220 | gene2243 | 3.59 | 2.12E-22 | chromate transporter |
| CPZ21_06395 | gene1278 | 3.57 | 1.57E-14 | phenol soluble modulin beta 1 |
| CPZ21_02870 | gene573 | 3.52 | 8.19E-23 | DNA-binding response regulator |
| CPZ21_10910 | gene2181 | 3.45 | 2.44E-19 | alpha-ketoacid dehydrogenase subunit beta |
| CPZ21_06375 | gene1274 | 3.43 | 5.67E-08 | beta-class phenol-soluble modulin |
| CPZ21_02860 | gene571 | 3.40 | 2.01E-21 | transporter |
| CPZ21_05795 | gene1158 | 3.34 | 4.59E-21 | 2-oxoacid ferredoxin oxidoreductase subunit beta |
| dhal | gene2162 | 3.25 | 3.57E-20 | dihydroxyacetone kinase subunit L |
| CPZ21_05800 | gene1159 | 3.21 | 8.53E-20 | 2-oxoacid:acceptor oxidoreductase, alpha subunit |
| CPZ21_10940 | gene2187 | 3.14 | 3.53E-18 | rhodanese domain-containing protein |
| ahpC | gene1991 | 3.11 | 8.45E-19 | peroxiredoxin |
| CPZ21_09965 | gene1992 | 3.09 | 1.13E-18 | alkyl hydroperoxide reductase subunit F |
| CPZ21_11165 | gene2232 | 3.08 | 1.77E-18 | Organic hydroperoxide resistance protein-like 2 |
| CPZ21_00655 | gene130 | 3.07 | 1.77E-18 | membrane protein |
| CPZ21_10820 | gene2163 | 3.07 | 2.13E-18 | PTS-dependent dihydroxyacetone kinase phosphotransferase subunit DhaM |
| CPZ21_11430 | gene2285 | 3.06 | 2.59E-18 | NAD(P)-dependent oxidoreductase |
| CPZ21_09675 | gene1934 | 3.05 | 5.88E-18 | putative sulfate exporter family transporter |
| CPZ21_10905 | gene2180 | 3.00 | 1.66E-14 | ABC transporter substrate-binding protein |
| lpgA | gene2179 | 2.98 | 5.98E-16 | dihydrolipoyl dehydrogenase |
| CPZ21_10810 | gene2161 | 2.97 | 1.63E-17 | dihydroxyacetone kinase subunit DhaK |
| CPZ21_03425 | gene684 | 2.75 | 5.17E-5 | competence protein ComK |
| CPZ21_08775 | gene1754 | 2.67 | 8.18E-14 | membrane protein |
| CPZ21_09700 | gene1939 | 2.67 | 2.56E-14 | LysR family transcriptional regulator |
| CPZ21_02825 | gene564 | 2.66 | 1.40E-14 | non-heme ferritin |
| CPZ21_08495 | gene1698 | 2.65 | 2.73E-14 | DUF1361 domain-containing protein |
| CPZ21_05510 | gene1101 | 2.59 | 5.30E-14 | SMC family ATPase |
| CPZ21_10805 | gene2160 | 2.54 | 1.20E-13 | glycerol dehydrogenase |
| CPZ21_04270 | gene853 | 2.46 | 7.52E-13 | DNA polymerase III subunit delta |
| CPZ21_10185 | gene2036 | 2.43 | 6.52E-06 | ABC transporter ATP-binding protein |
| CPZ21_03965 | gene792 | 2.43 | 6.38E-12 | DUF4930 domain-containing protein |
| CPZ21_04705 | gene940 | 2.38 | 7.89E-11 | short-chain dehydrogenase |
| CPZ21_05185 | gene1036 | 2.37 | 3.07E-12 | toxic anion resistance protein TelA |
| CPZ21_05515 | gene1102 | 2.30 | 2.43E-11 | exonuclease SbcCD subunit D |
| CPZ21_08505 | gene1709 | 2.28 | 2.29E-11 | undecaprenyl-diphosphatase |
| arcC | gene2154 | 2.26 | 1.54E-10 | carbamate kinase |
| CPZ21_04985 | gene996 | 2.26 | 1.69E-10 | ribonuclease H |
| CPZ21_11255 | gene2250 | 2.25 | 4.63E-11 | transcriptional regulator |
| CPZ21_09725 | gene1944 | 2.23 | 5.12E-11 | glycosyl transferase family 1 |
| CPZ21_10190 | gene2037 | 2.23 | 4.73E-08 | bacitracin ABC transporter permease |
| CPZ21_10950 | gene2189 | 2.23 | 5.79E-11 | polyisoprenoid-binding protein |
| CPZ21_00660 | gene131 | 2.19 | 1.00E-10 | glycerate kinase |
| CPZ21_02920 | gene583 | 2.18 | 2.05E-10 | glycosyl transferase |
| CPZ21_02820 | gene563 | 2.16 | 2.58E-09 | DNA polymerase III subunit epsilon |
| CPZ21_09660 | gene1931 | 2.15 | 3.08E-09 | spermidine N1-acetyltransferase |
| CPZ21_07655 | gene1530 | 2.13 | 2.95E-10 | SUF system NifU family Fe-S cluster assembly protein |
| CPZ21_04110 | gene821 | 2.08 | 8.15E-10 | LLM class flavin-dependent oxidoreductase |
| moaA | gene287 | 2.07 | 8.76E-10 | GTP 3',8-cyclase MoaA |
| CPZ21_09950 | gene1989 | 2.05 | 1.41E-09 | NADPH-dependent oxidoreductase |
| CPZ21_05190 | gene1037 | 2.05 | 1.13E-09 | 5-bromo-4-chloroindolyl phosphate hydrolysis protein |
| CPZ21_10915 | gene2182 | 2.01 | 4.55E-09 | 2-oxo acid dehydrogenase subunit E2 |
| moaD | gene285 | 2.01 | 3.11E-09 | molybdopterin converting factor subunit 1 |
| CPZ21_01425 | gene284 | 2.00 | 2.84E-09 | molybdenum cofactor biosynthesis protein MoaE |
| CPZ21_01435 | gene286 | 1.99 | 3.54E-09 | molybdenum cofactor guanylyltransferase MobA |
| CPZ21_01455 | gene290 | 1.97 | 5.58E-06 | transcriptional regulator, MarR family |
| CPZ21_07675 | gene1534 | 1.96 | 5.39E-09 | DUF368 domain-containing protein |
| CPZ21_09030 | gene1805 | 1.95 | 6.19E-09 | heme-binding protein |
| CPZ21_04440 | gene887 | 1.89 | 1.56E-08 | superoxide dismutase |
| CPZ21_10225 | gene2044 | 1.89 | 3.4 E-03 | XRE family transcriptional regulator |
| CPZ21_08225 | gene1644 | 1.88 | 1.91E-08 | glycerate kinase |
| CPZ21_10230 | gene2045 | 1.88 | 5.77E-08 | TetR/AcrR family transcriptional regulator |
| CPZ21_07885 | gene1576 | 1.85 | 1.68E-05 | DUF1433 domain-containing protein |
| CPZ21_09655 | gene1930 | 1.82 | 8.84E-08 | lysine decarboxylase |
| CPZ21_00825 | gene164 | 1.78 | 1.12E-07 | DUF3139 domain-containing protein |
| CPZ21_06685 | gene1336 | 1.77 | 1.22E-07 | cell-wall-binding lipoprotein |
| CPZ21_05195 | gene1038 | 1.77 | 1.51E-07 | acylphosphatase |
| CPZ21_00310 | gene61 | 1.77 | 1.26E-07 | aldehyde dehydrogenase |
| CPZ21_01855 | gene370 | 1.76 | 1.28E-07 | MFS transporter |
| sufC | gene1532 | 1.76 | 1.25E-07 | Fe-S cluster assembly protein SufD |
| CPZ21_03565 | gene712 | 1.76 | 1.32E-07 | M42 family peptidase |
| CPZ21_07660 | gene1531 | 1.75 | 1.54E-07 | cysteine desulfurase |
| CPZ21_03570 | gene713 | 1.74 | 2.14E-07 | thioredoxin |
| CPZ21_02815 | gene562 | 1.74 | 5.19E-07 | DNA polymerase IV |
| CPZ21_02265 | gene452 | 1.69 | 3.71E-07 | D-ala D-ala ligase N-terminal domain protein |
| CPZ21_11190 | gene2237 | 1.68 | 1.2 E-04 | PTS mannose transporter subunit IIABC |
| CPZ21_01275 | gene254 | 1.68 | 4.23E-07 | CHAP domain-containing protein |
| sufB | gene1529 | 1.67 | 4.75E-07 | Fe-S cluster assembly protein SufB |
| CPZ21_10550 | gene2019 | 1.66 | 5.91E-07 | acetyl-CoA C-acetyltransferase |
| CPZ21_07545 | gene1508 | 1.66 | 2.15E-06 | hypothetical protein |
| sufC | gene1533 | 1.66 | 6.38E-07 | Fe-S cluster assembly ATPase SufC |
| CPZ21_00705 | gene140 | 1.65 | 6.59E-07 | aminoacyltransferase |
| CPZ21_09045 | gene1808 | 1.65 | 6.95E-07 | APC family permease |
| CPZ21_00165 | gene32 | 1.63 | 1.05E-06 | alpha/beta hydrolase |
| CPZ21_03495 | gene698 | 1.62 | 1.16E-06 | TIGR01212 family radical SAM protein |
| CPZ21_01520 | gene303 | 1.62 | 1.11E-06 | hypothetical protein |
| CPZ21_04725 | gene944 | 1.62 | 1.18E-06 | site-specific tyrosine recombinase XerD |
| CPZ21_03395 | gene678 | 1.58 | 2.05E-06 | aldo/keto reductase |
| CPZ21_04720 | gene943 | 1.58 | 2.24E-06 | transcriptional repressor |
| CPZ21_06865 | gene1372 | 1.58 | 2.49E-06 | methicillin resistance protein FmtA |
| CPZ21_09650 | gene1929 | 1.57 | 3.04E-06 | dTMP kinase |
| argF | gene2155 | 1.57 | 5.88E-06 | ornithine carbamoyltransferase |
| CPZ21_09500 | gene1899 | 1.55 | 3.05E-06 | Hsp33 family molecular chaperone HslO |
| CPZ21_01925 | gene384 | 1.54 | 3.52E-06 | Cof-type HAD-IIB family hydrolase |
| dltD | gene1512 | 1.54 | 3.77E-06 | D-alanyl-lipoteichoic acid biosynthesis protein DltD |
| CPZ21_08490 | gene1697 | 1.52 | 5.87E-06 | multidrug efflux MFS transporter NorA |
| CPZ21_10235 | gene2046 | 1.52 | 1.58E-05 | DUF3147 domain-containing protein |
| CPZ21_08750 | gene1749 | 1.52 | 4.61E-06 | glycosyltransferase family 2 protein |
| CPZ21_10560 | gene2111 | 1.51 | 0.000555302 | staphostatin A |
| CPZ21_00630 | gene125 | 1.51 | 1.52E-05 | APC family permease |
| CPZ21_04565 | gene912 | 1.49 | 7.06E-06 | aminopeptidase P family protein |
| CPZ21_07300 | gene1459 | 1.49 | 2.04E-05 | competence protein |
| CPZ21_08755 | gene1750 | 1.49 | 7.15E-06 | CDP-glycerol glycerophosphotransferase family protein |
| CPZ21_07020 | gene1403 | 1.48 | 1.60E-05 | IDEAL domain-containing protein |
| CPZ21_08235 | gene1646 | 1.47 | 1.25E-05 | EMYY motif lipoprotein |
| CPZ21_07460 | gene1491 | 1.46 | 1.08E-05 | NADH-dependent flavin oxidoreductase |
| CPZ21_00650 | gene129 | 1.45 | 1.18E-05 | MFS transporter |
| CPZ21_09000 | gene1799 | 1.45 | 2.70E-05 | DUF1450 domain-containing protein |
| CPZ21_07710 | gene1541 | 1.44 | 1.87E-05 | toprim domain protein |
| mobB | gene283 | 1.44 | 1.50E-05 | molybdopterin-guanine dinucleotide biosynthesis protein B |
| dltB | gene1514 | 1.43 | 1.90E-05 | D-alanyl-lipoteichoic acid biosynthesis protein DltB |
| CPZ21_06745 | gene1348 | 1.42 | 1.75E-05 | hypothetical protein |
| fecD | gene361 | 1.42 | 2.25E-05 | iron-dicitrate ABC transporter permease |
| CPZ21_10790 | gene2157 | 1.41 | 2.31E-03 | FosB family fosfomycin resistance bacillithiol transferase |
| CPZ21_11180 | gene2235 | 1.40 | 2.15E-05 | YhgE/Pip domain-containing protein |
| mraW | gene697 | 1.40 | 2.58E-05 | methyltransferase domain-containing protein |
| CPZ21_03265 | gene652 | 1.40 | 2.29E-05 | cadmium transporter |
| CPZ21_09670 | gene1933 | 1.39 | 0.0139 | transposase, IS116/IS110/IS902 family |
| CPZ21_04875 | gene974 | 1.38 | 2.70E-05 | zinc metallopeptidase |
| CPZ21_03445 | gene688 | 1.38 | 2.81E-05 | autolysin |
| thiO | gene70 | 1.38 | 1.95E-04 | glycine oxidase ThiO |
| CPZ21_00345 | gene68 | 1.38 | 1.95E-04 | thiazole synthase |
| CPZ21_09370 | gene1873 | 1.37 | 3.36E-05 | ATP-dependent Clp protease ATP-binding subunit |
| CPZ21_00155 | gene30 | 1.36 | 4.24E-05 | thioredoxin |
| CPZ21_03235 | gene646 | 1.36 | 3.58E-05 | peptidyl-prolyl cis-trans isomerase |
| CPZ21_07705 | gene1540 | 1.36 | 8.36E-05 | thioredoxin |
| CPZ21_00860 | gene171 | 1.36 | 6.54E-05 | magnesium transporter CorA family protein |
| metE | gene2011 | 1.35 | 5.74E-05 | mechanosensitive ion channel family protein |
| CPZ21_01760 | gene351 | 1.34 | 4.54E-05 | zinc-binding alcohol dehydrogenase family protein |
| CPZ21_07310 | gene1461 | 1.33 | 5.17E-05 | transcriptional regulator Spx |
| CPZ21_02945 | gene588 | 1.33 | 5.72E-05 | metal-dependent hydrolase |
| CPZ21_06630 | gene1325 | 1.33 | 5.80E-05 | DUF4064 domain-containing protein |
| CPZ21_04710 | gene941 | 1.33 | 5.91E-05 | aldo/keto reductase |
| CPZ21_06755 | gene1350 | 1.32 | 6.58E-05 | oleate hydratase |
| CPZ21_11450 | gene2289 | 1.32 | 6.46E-05 | NAD dependent epimerase/dehydratase |
| CPZ21_08135 | gene1626 | 1.32 | 6.39E-05 | excinuclease ABC subunit UvrA |
| CPZ21_03500 | gene699 | 1.31 | 7.42E-05 | MFS transporter |
| CPZ21_08440 | gene1687 | 1.30 | 1.01E-04 | glycosyltransferase |
| CPZ21_07820 | gene1563 | 1.30 | 1.01E-04 | cation transporter |
| CPZ21_05720 | gene1143 | 1.30 | 8.39E-05 | glutathione peroxidase |
| nagB | gene1822 | 1.30 | 1.58E-04 | glucosamine-6-phosphate deaminase |
| CPZ21_05015 | gene1002 | 1.30 | 9.41E-05 | ABC transporter ATP-binding protein |
| CPZ21_11170 | gene2233 | 1.30 | 9.01E-05 | cell wall surface anchor protein |
| CPZ21_05010 | gene1001 | 1.30 | 9.52E-05 | ABC transporter permease |
| CPZ21_09375 | gene1874 | 1.29 | 8.79E-05 | protein arginine kinase |
| CPZ21_03535 | gene706 | 1.29 | 8.57E-05 | YtxH domain-containing protein |
| CPZ21_00265 | gene52 | 1.29 | 9.04E-05 | NAD(P)H-dependent oxidoreductase |
| CPZ21_03660 | gene731 | 1.29 | 4.30E-03 | osmotically inducible protein C |
| CPZ21_06960 | gene1391 | 1.28 | 5.87E-04 | NINE protein |
| CPZ21_03520 | gene703 | 1.28 | 1.32E-04 | NAD(P)/FAD-dependent oxidoreductase |
| CPZ21_05005 | gene1000 | 1.27 | 1.17E-04 | BrxA/BrxB family bacilliredoxin |
| CPZ21_07030 | gene1405 | 1.27 | 1.22E-04 | DUF2187 domain-containing protein |
| CPZ21_08140 | gene1627 | 1.27 | 1.22E-03 | excinuclease ABC subunit B |
| CPZ21_10555 | gene2110 | 1.26 | 8.97E-04 | YSIRK signal domain/LPXTG anchor domain surface protein, partial |
| CPZ21_02635 | gene526 | 1.26 | 1.73E-04 | choloylglycine hydrolase |
| CPZ21_04790 | gene957 | 1.26 | 1.26E-04 | thioredoxin reductase |
| CPZ21_08360 | gene1671 | 1.22 | 1.93E-04 | LTA synthase family protein |
| CPZ21_07355 | gene1470 | 1.22 | 1.97E-04 | ketoacyl-ACP synthase III |
| CPZ21_05930 | gene1185 | 1.22 | 2.02E-04 | proline--tRNA ligase |
| lepB | gene1482 | 1.22 | 2.25E-04 | signal peptidase I |
| CPZ21_00125 | gene24 | 1.22 | 2.15E-04 | LrgB family protein |
| CPZ21_02695 | gene538 | 1.21 | 2.35E-04 | lactonase family protein |
| manA | gene2236 | 1.21 | 0.0121 | mannose-6-phosphate isomerase, class I |
| CPZ21_11135 | gene2226 | 1.21 | 6.64E-04 | peptide-methionine (S)-S-oxide reductase |
| CPZ21_02715 | gene542 | 1.21 | 3.55E-04 | pectate lyase |
| CPZ21_03260 | gene651 | 1.20 | 2.66E-04 | multidrug ABC transporter ATP-binding protein |
| CPZ21_01470 | gene293 | 1.20 | 2.67E-04 | AcrB/AcrD/AcrF family protein |
| CPZ21_02270 | gene453 | 1.19 | 2.81E-04 | UDP-N-acetylmuramoyl-tripeptide--D-alanyl-D-alanine ligase |
| CPZ21_03255 | gene650 | 1.19 | 3.22E-04 | ABC transporter ATP-binding protein |
| CPZ21_01980 | gene395 | 1.19 | 4.01E-03 | DUF393 domain-containing protein |
| CPZ21_01185 | gene236 | 1.18 | 3.29E-04 | amino acid permease |
| dltC | gene1513 | 1.18 | 6.84E-04 | D-alanine--poly(phosphoribitol) ligase subunit 2 |
| CPZ21_04995 | gene998 | 1.18 | 3.70E-04 | zinc-finger domain-containing protein |
| CPZ21_01785 | gene356 | 1.18 | 5.27E-04 | MFS transporter |
| CPZ21_01795 | gene358 | 1.17 | 4.50E-04 | alanine racemase |
| CPZ21_06610 | gene1321 | 1.17 | 3.59E-04 | inositol monophosphatase family protein |
| CPZ21_01150 | gene229 | 1.17 | 3.84E-04 | MurR/RpiR family transcriptional regulator |
| CPZ21_03530 | gene705 | 1.16 | 3.98E-04 | rRNA pseudouridine synthase |
| rarD | gene2176 | 1.16 | 5.15E-04 | rarD protein |
| CPZ21_07420 | gene1483 | 1.16 | 6.25E-04 | TVP38/TMEM64 family protein |
| rseP | gene1186 | 1.15 | 4.73E-04 | RIP metalloprotease RseP |
| CPZ21_01750 | gene349 | 1.14 | 6.54E-04 | LLM class flavin-dependent oxidoreductase |
| CPZ21_10565 | gene2112 | 1.14 | 1.65E-04 | cysteine protease |
| CPZ21_01790 | gene357 | 1.13 | 6.49E-04 | siderophore synthetase |
| CPZ21_07585 | gene1516 | 1.13 | 0.00213 | teichoic acid D-Ala incorporation-associated protein DltX |
| phnC | gene2213 | 1.13 | 0.00967 | phosphonates import ATP-binding protein PhnC |
| CPZ21_01755 | gene350 | 1.13 | 5.72E-04 | NADP-dependent oxidoreductase |
| CPZ21_08255 | gene1650 | 1.13 | 7.71E-04 | iron ABC transporter substrate-binding protein |
| CPZ21_05640 | gene1127 | 1.13 | 6.38E-04 | Thermonuclease |
| CPZ21_10550 | gene2109 | 1.12 | 0.00187 | YSIRK signal domain/LPXTG anchor domain surface protein, partial |
| lepB | gene1481 | 1.11 | 7.46E-04 | signal peptidase I |
| CPZ21_04265 | gene852 | 1.11 | 0.00288 | DNA internalization-related competence protein ComEC/Rec2 |
| thiS | gene69 | 1.11 | 0.00585 | thiamine biosynthesis protein ThiS |
| CPZ21_11365 | gene2272 | 1.10 | 0.00156 | TIGR01741 family protein |
| CPZ21_00245 | gene48 | 1.10 | 0.00263 | N-acetyltransferase |
| CPZ21_02975 | gene594 | 1.09 | 0.000944 | thioredoxin-dependent thiol peroxidase |
| CPZ21_01120 | gene223 | 1.08 | 0.00355 | amidohydrolase |
| dltA | gene1515 | 1.07 | 0.00112 | D-alanine--poly(phosphoribitol) ligase subunit 1 |
| CPZ21_10770 | gene2153 | 1.07 | 0.00142 | YfcC family protein |
| CPZ21_11715 | gene2342 | 1.07 | 0.01318 | 2-dehydropantoate 2-reductase |
| CPZ21_11050 | gene2209 | 1.06 | 0.00371 | MFS transporter |
| gtfA | gene2223 | 1.06 | 0.00141 | accessory Sec system glycosyltransferase GtfA |
| CPZ21_11275 | gene2254 | 1.05 | 0.00136 | pyruvate decarboxylase |
| CPZ21_04260 | gene851 | 1.05 | 0.00156 | ComE operon protein 2 |
| CPZ21_08264 | gene1651 | 1.05 | 0.00164 | iron ABC transporter ATP-binding protein |
| CPZ21_00905 | gene180 | 1.05 | 0.00136 | teichoic acid biosynthesis TagF |
| CPZ21_01770 | gene353 | 1.04 | 0.00144 | DUF2273 domain-containing protein |
| CPZ21_08955 | gene1793 | 1.04 | 0.00157 | YwhD family protein |
| CPZ21_06450 | gene1289 | 1.04 | 0.00153 | endonuclease MutS2 |
| CPZ21_11360 | gene2271 | 1.04 | 0.003519 | TIGR01741 family protein |
| CPZ21_01780 | gene355 | 1.04 | 0.00158 | siderophore biosynthesis protein, IucA/IucC family |
| CPZ21_02070 | gene413 | 1.04 | 0.00154 | aldehyde dehydrogenase family protein |
| gtfB | gene2224 | 1.04 | 0.00174 | accessory Sec system glycosylation chaperone GtfB |
| CPZ21_06710 | gene1341 | 1.03 | 0.00173 | cytochrome D ubiquinol oxidase subunit II |
| CPZ21_00635 | gene126 | 1.02 | 0.00195 | amino acid permease |
| thiF | gene67 | 1.02 | 0.00338 | thiazole biosynthesis adenylyltransferase ThiF |
| CPZ21_05545 | gene1108 | 1.02 | 0.00258 | DUF896 family protein |
| CPZ21_00095 | gene18 | 1.02 | 0.00193 | hydroxymethylglutaryl-CoA synthase |
| CPZ21_10055 | gene2010 | 1.01 | 0.00276 | DUF951 domain-containing protein |
| CPZ21_02985 | gene596 | 1.00 | 0.00222 | peroxide-responsive repressor PerR |
| CPZ21_00325 | gene64 | 1.00 | 0.00223 | sodium:proton antiporter |
| CPZ21_06640 | gene1327 | 1.00 | 0.00462 | ABC transporter permease |

**Table 3S.** 10 binding modes of aloe-emodin on LPS and their total binding energy

| Binding Mode | Minimization Iter Energy (kJ/mol) | Total Energy (kJ/mol) |
| --- | --- | --- |
| B1 | -1355.290 | -1358.7612 |
| B2 | -1382.156 | -1386.1200 |
| B3 | -1377.957 | -1382.0778 |
| B4 | -1353.377 | -1356.8575 |
| B5 | -1379.919 | -1385.3333 |
| B6 | -1377.334 | -1381.2596 |
| B7 | -1399.261 | -1403.2787 |
| B8 | -1421.901 | -1425.7789 |
| B9 | -1399.537 | -1403.3081 |
| B10 | -1412.212 | -1416.0759 |

**Table 4S** 14 binding modes of aloe-emodin on peptidoglycan and their total binding energy

| Binding Mode | Minimization Iter Energy (kJ/mol) | Total Energy (kJ/mol) |
| --- | --- | --- |
| B1 | -1883.836 | -1883.6765 |
| B2 | -1863.353 | -1863.1161 |
| B3 | -1875.350 | -1874.4182 |
| B4 | -1879.281 | -1878.3604 |
| B5 | -1906.079 | -1905.2155 |
| B6 | -1944.408 | -1945.2531 |
| B7 | -1871.816 | -1870.8873 |
| B8 | -1879.906 | -1879.2258 |
| B9 | -1879.371 | -1878.7255 |
| B10 | -1899.853 | -1898.8177 |
| B11 | -1853.330 | -1853.4219 |
| B12 | -1852.021 | -1851.1982 |
| B13 | -1850.649 | -1849.0618 |
| B14 | -1883.613 | -1882.9829 |

**Table 5S.** Primers used for RNAseq was validated by qRT-PCR.

| **Target gene** | **Primers sequence (5’ to 3’)** | | **Amplicon (bp)** |
| --- | --- | --- | --- |
| 16S r RNA | F | GGGCTACACACGTGCTACAA | 176 |
|  | R | GTACAAGACCCGGGAACGTA |  |
| gpmA | F | CGTCCGCCTAGAGAAAGTGA | 134 |
|  | R | GTCCAGAACGGAACAACTCG |  |
| CPZ21-03630 | F | CGACAATTGCGGGAGAAACG | 175 |
|  | R | GGAGCACTTCTCGTCTTAGGT |  |
| CPZ21_05265 | F | ATGCTGCGCCTTTGAGTAAA | 83 |
|  | R | AGGACGTATTCGCCGAGATG |  |
| CPZ21_02470 | F | TATAGCCCTGTTGCCCACTG | 85 |
|  | R | GAACTACTGTTCCACCCGCA |  |
| CPZ21_00595  (IrgA) | F | AGCTGGCATTGGTATAGGCA | 147 |
|  | R | TTGGAACAAACGCATCTCGT |  |
| CPZ21_10075 | F | TCTTCGCATAGAGCGTTCCC | 103 |
|  | R | CATGCCTGTTCGTCCGCTAT |  |
| CPZ21_10160, (sdrF) | F | TGTGACTGTTGTTTTCGTGCC | 88 |
|  | R | TGAGACACCGGAAGGCTACA |  |
